# Supplementary figures and images for: Hepatic conversion of acetyl-CoA to acetate plays crucial roles in energy stress (part 1 of 2)
Source: eLife. 2023 Oct 30;12:RP87419. doi: 10.7554/eLife.87419 (PMC10615369; doi:10.7554/eLife.87419)

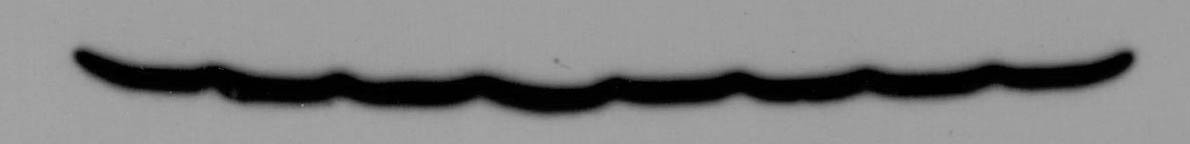

Supplement: Figure 3—source data 1. [file elife-87419-fig3-data1.zip › Figure 3-source data 1/3B/20201024-WB/actin.jpg]

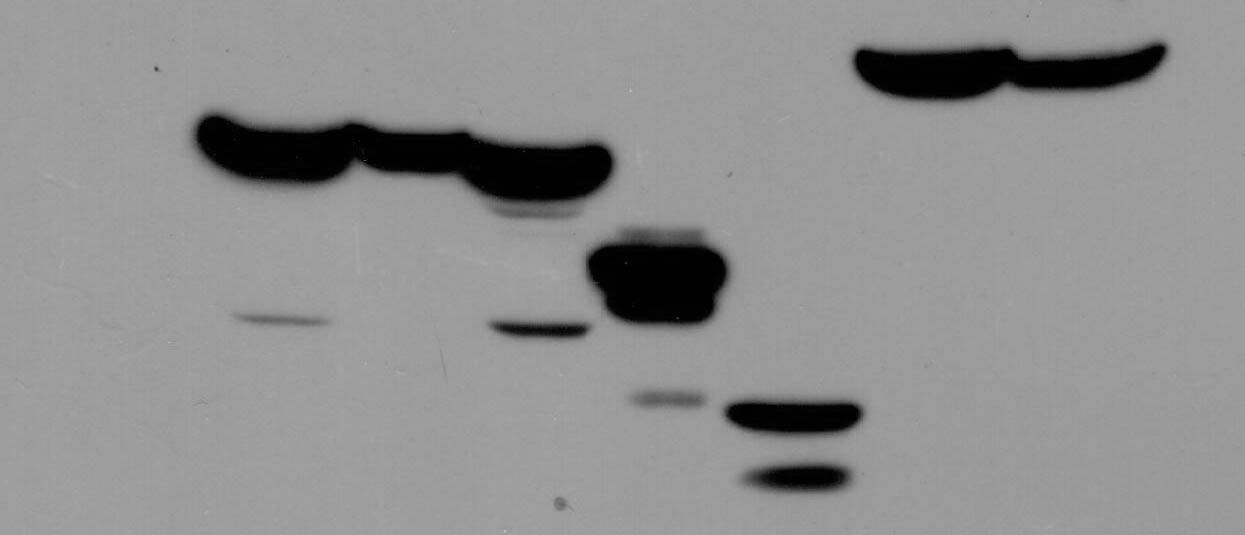

Supplement: Figure 3—source data 1. [file elife-87419-fig3-data1.zip › Figure 3-source data 1/3B/20201024-WB/flag.jpg]

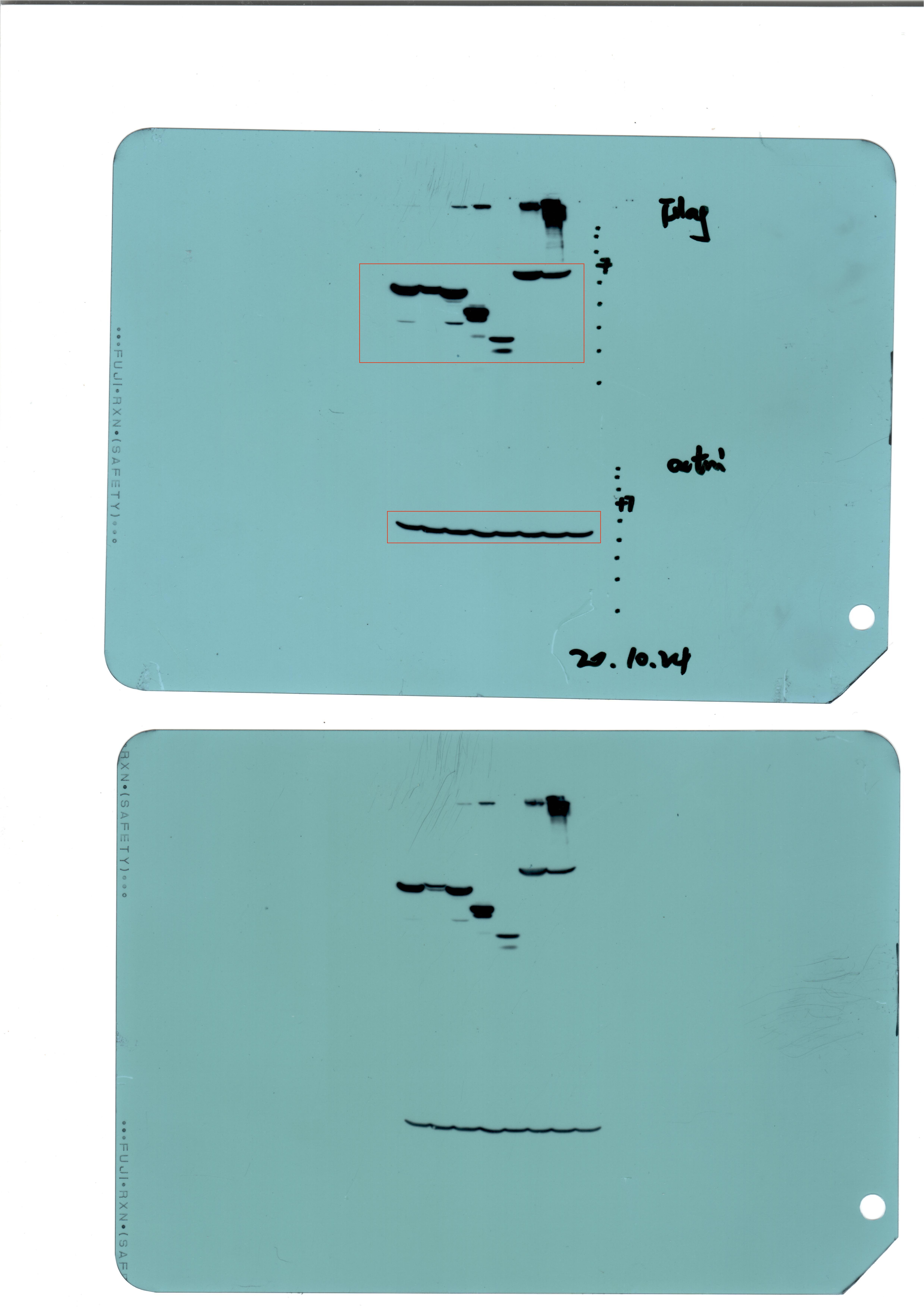

Supplement: Figure 3—source data 1. [file elife-87419-fig3-data1.zip › Figure 3-source data 1/3B/20201024-WB/Scan.jpg]

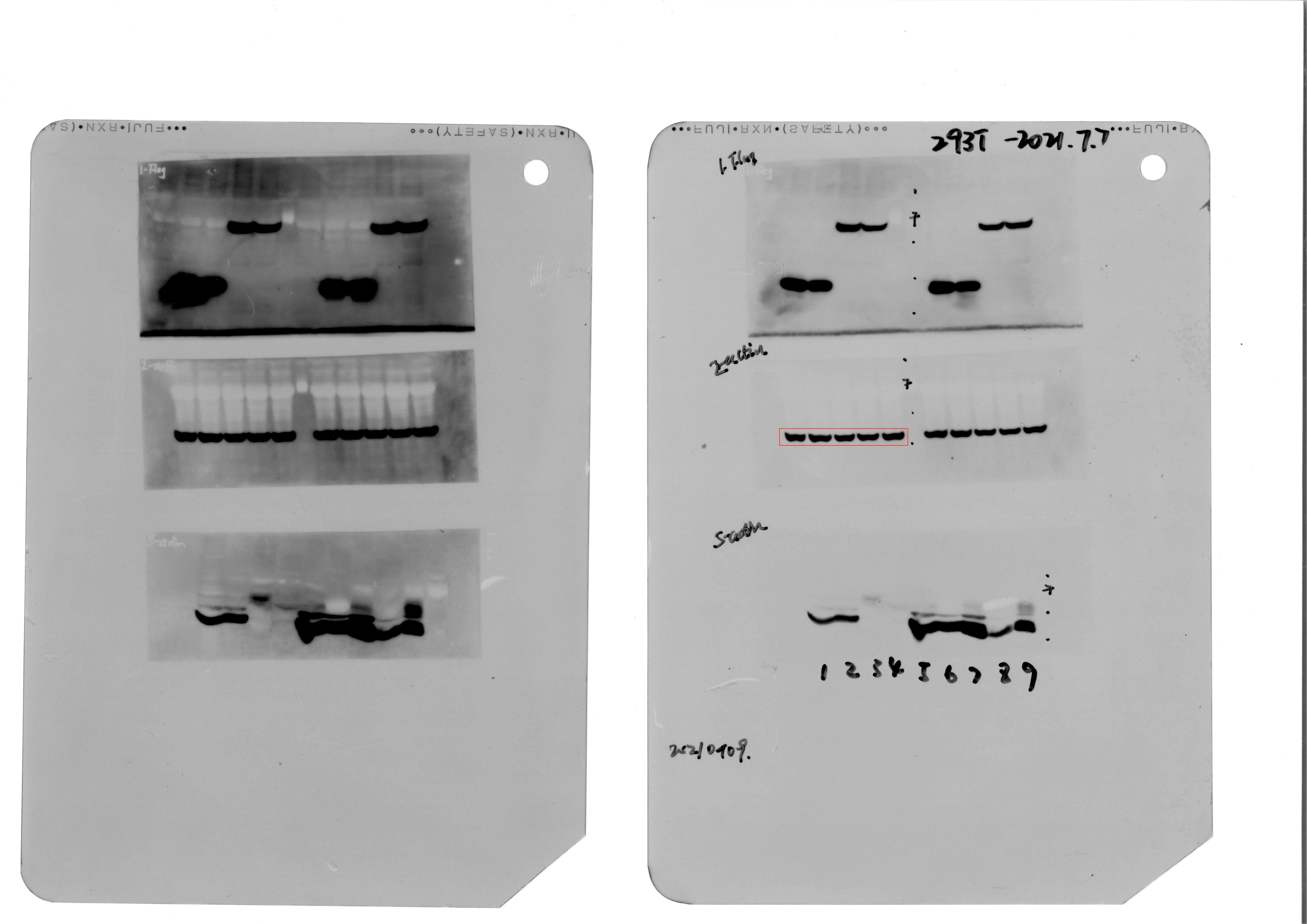

Supplement: Figure 3—source data 1. [file elife-87419-fig3-data1.zip › Figure 3-source data 1/3C/20210707-WB/1.jpg]

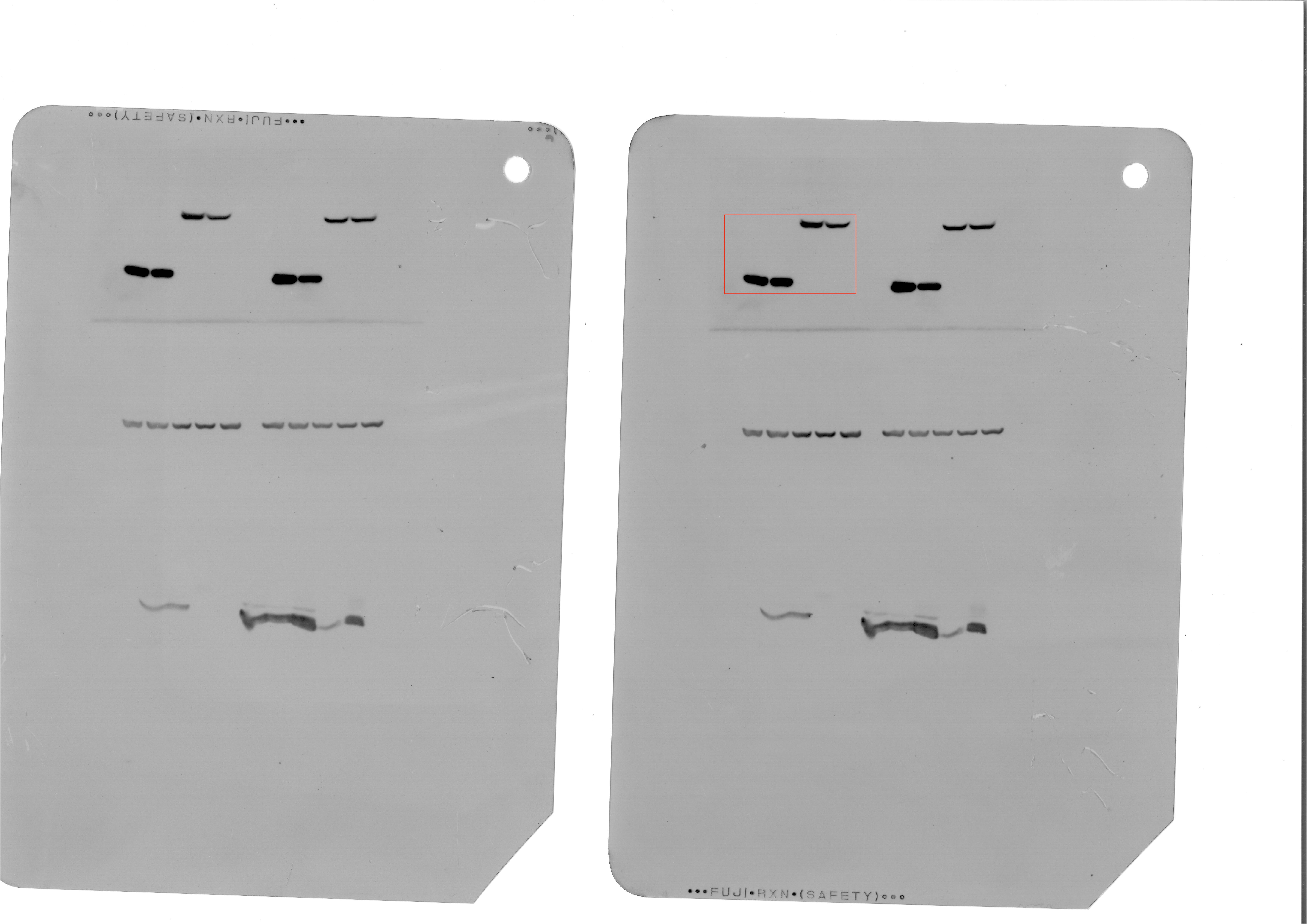

Supplement: Figure 3—source data 1. [file elife-87419-fig3-data1.zip › Figure 3-source data 1/3C/20210707-WB/2.jpg]

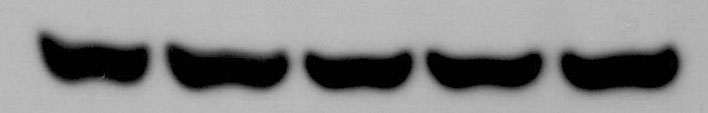

Supplement: Figure 3—source data 1. [file elife-87419-fig3-data1.zip › Figure 3-source data 1/3C/20210707-WB/actin.jpg]

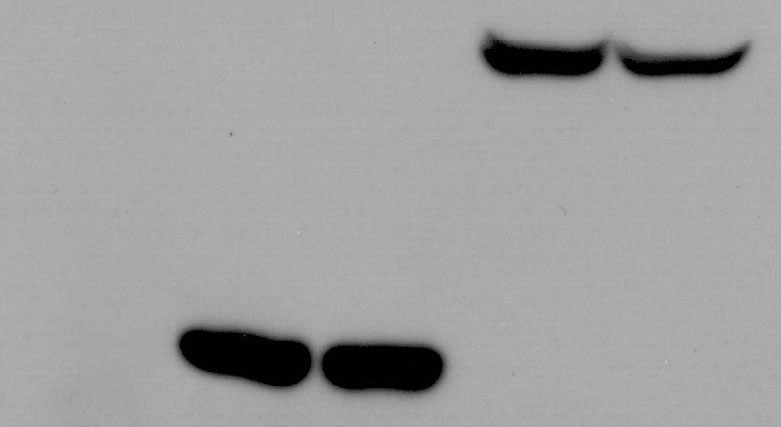

Supplement: Figure 3—source data 1. [file elife-87419-fig3-data1.zip › Figure 3-source data 1/3C/20210707-WB/flag.1.jpg]

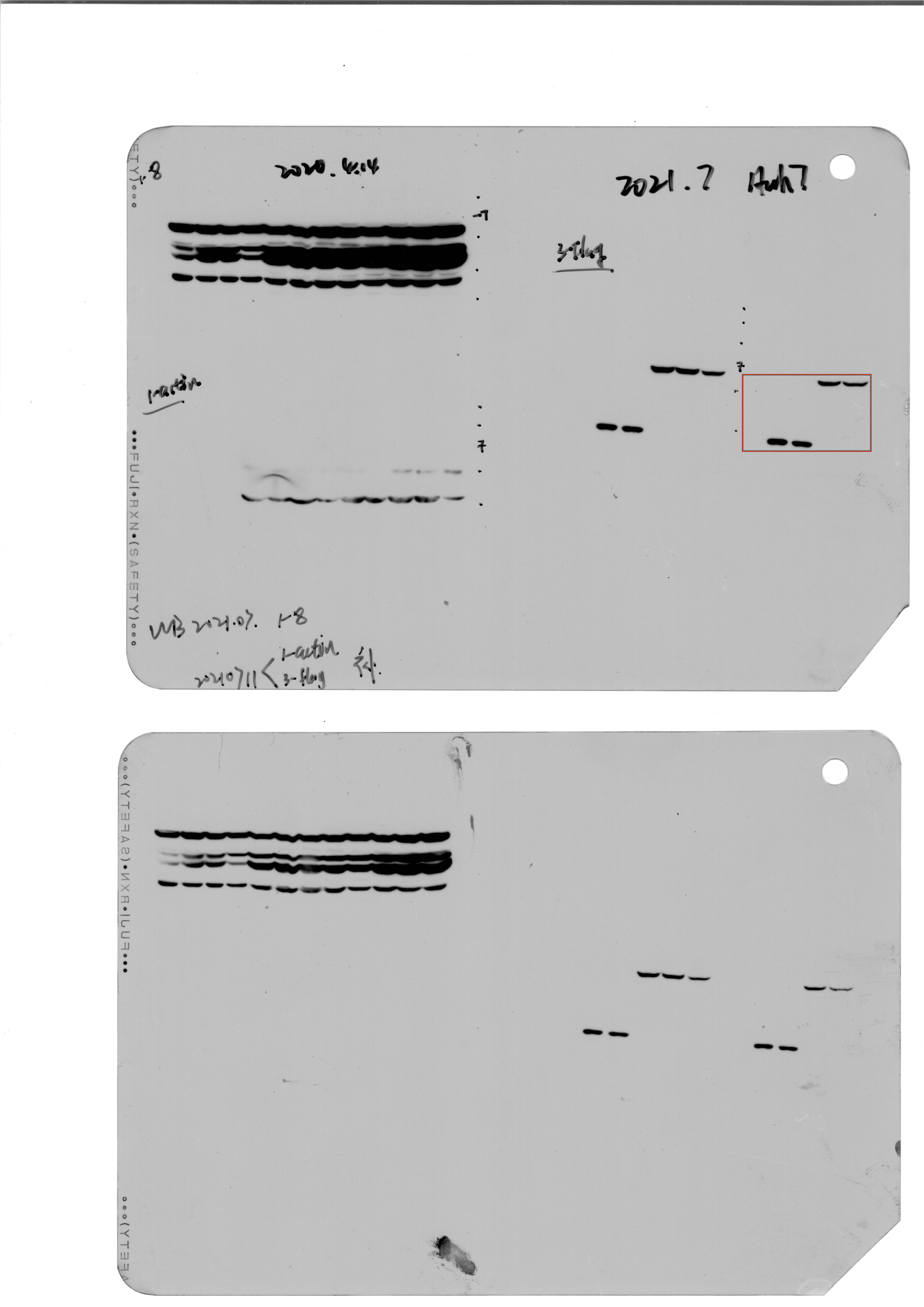

Supplement: Figure 3—source data 1. [file elife-87419-fig3-data1.zip › Figure 3-source data 1/3D/20210711-WB/1.jpg]

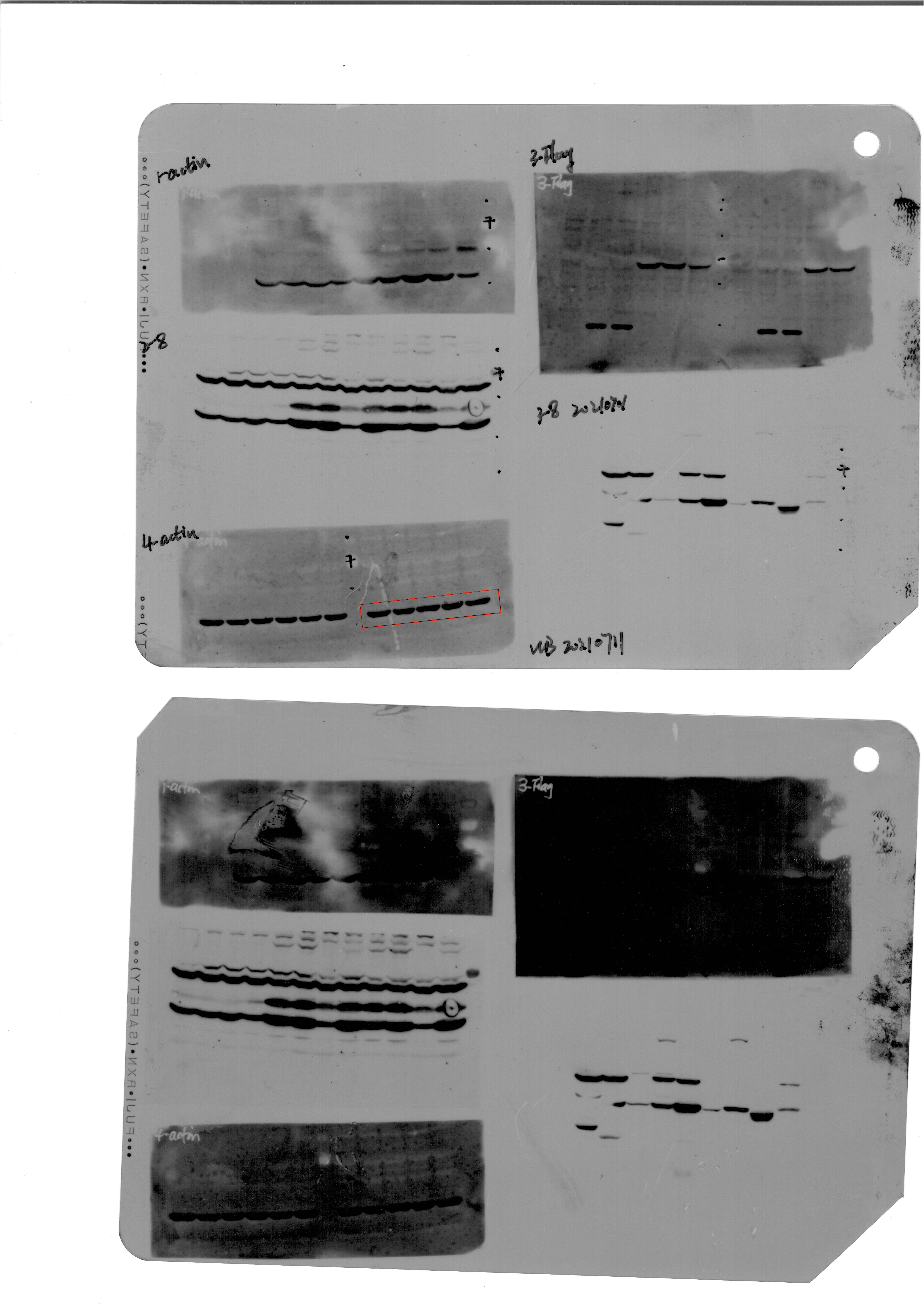

Supplement: Figure 3—source data 1. [file elife-87419-fig3-data1.zip › Figure 3-source data 1/3D/20210711-WB/3.jpg]

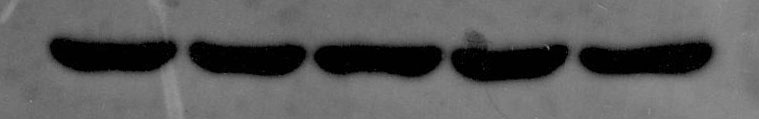

Supplement: Figure 3—source data 1. [file elife-87419-fig3-data1.zip › Figure 3-source data 1/3D/20210711-WB/actin.jpg]

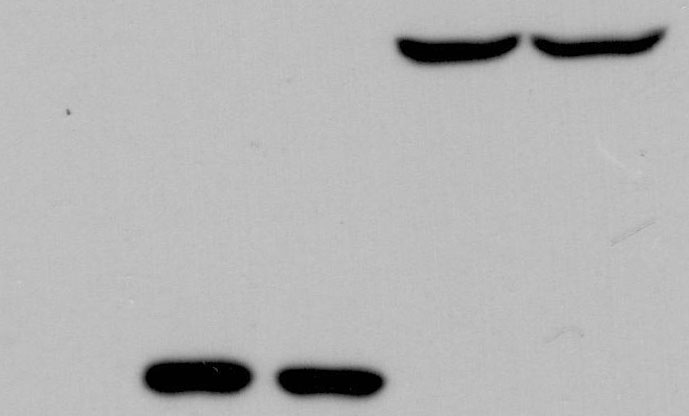

Supplement: Figure 3—source data 1. [file elife-87419-fig3-data1.zip › Figure 3-source data 1/3D/20210711-WB/Flag.jpg]

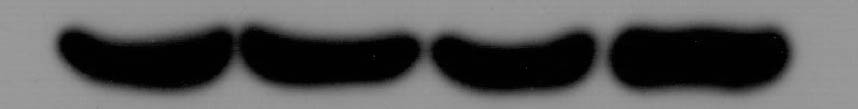

Supplement: Figure 3—source data 1. [file elife-87419-fig3-data1.zip › Figure 3-source data 1/3EF/20201114-WB/12-actin.jpg]

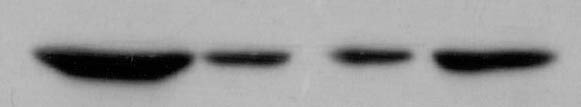

Supplement: Figure 3—source data 1. [file elife-87419-fig3-data1.zip › Figure 3-source data 1/3EF/20201114-WB/20201114-ACOT12.jpg]

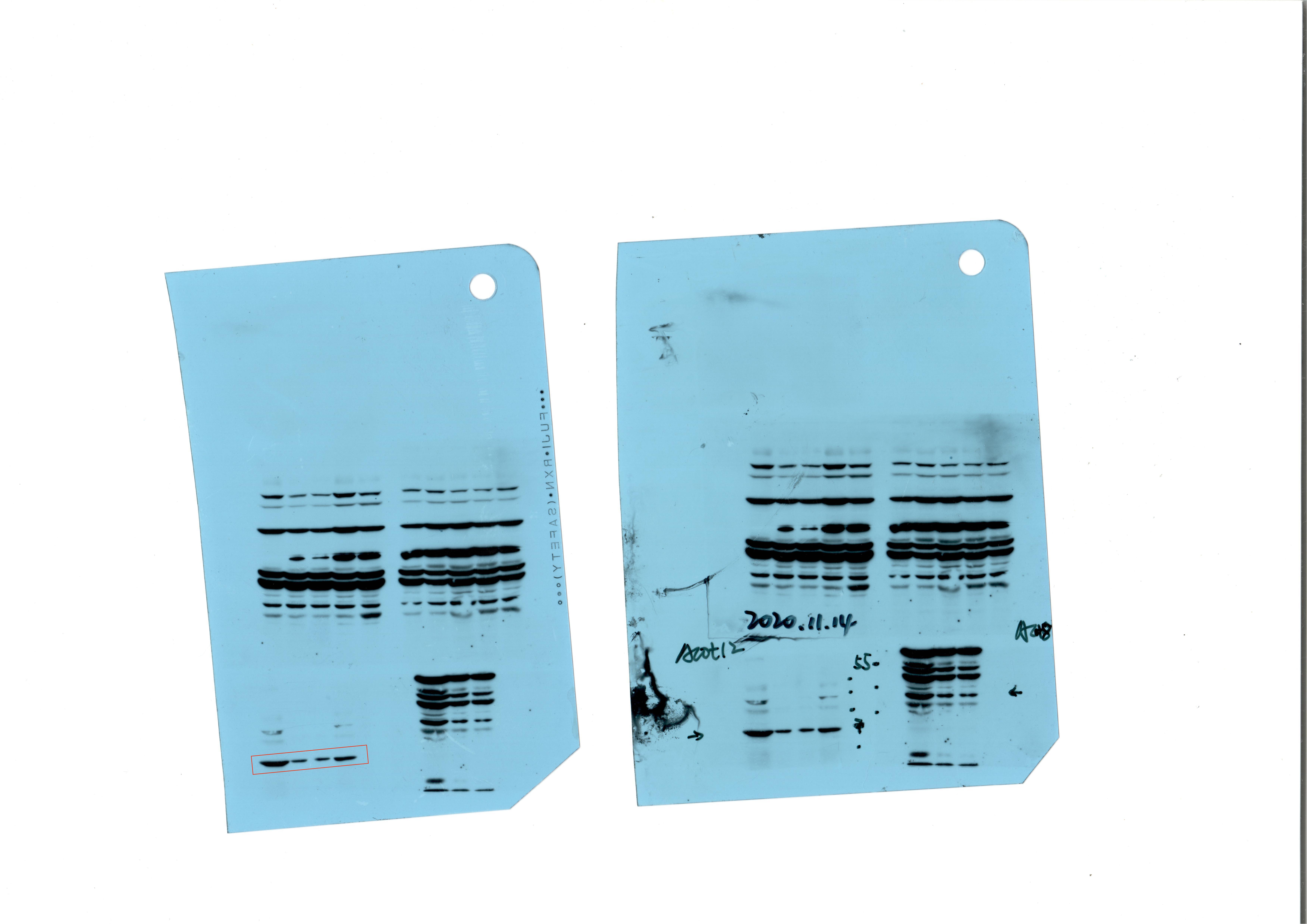

Supplement: Figure 3—source data 1. [file elife-87419-fig3-data1.zip › Figure 3-source data 1/3EF/20201114-WB/20201114.jpg]

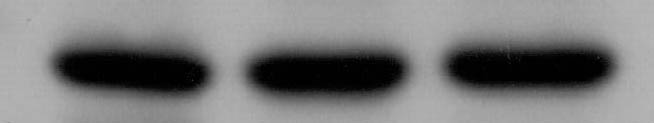

Supplement: Figure 3—source data 1. [file elife-87419-fig3-data1.zip › Figure 3-source data 1/3EF/20201114-WB/8-actin.jpg]

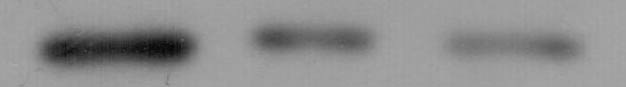

Supplement: Figure 3—source data 1. [file elife-87419-fig3-data1.zip › Figure 3-source data 1/3EF/20201114-WB/8.jpg]

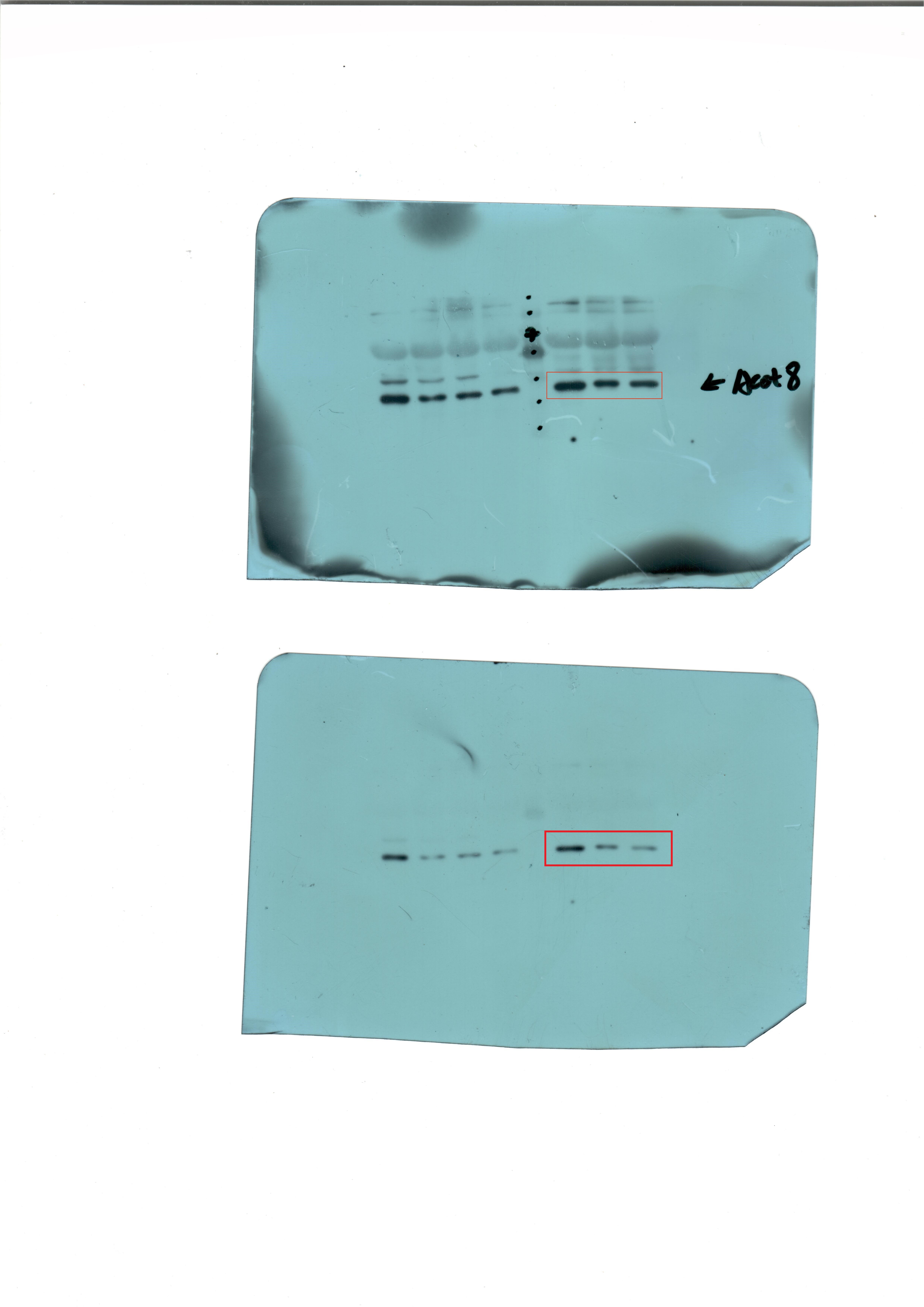

Supplement: Figure 3—source data 1. [file elife-87419-fig3-data1.zip › Figure 3-source data 1/3EF/20201114-WB/Scan1.jpg]

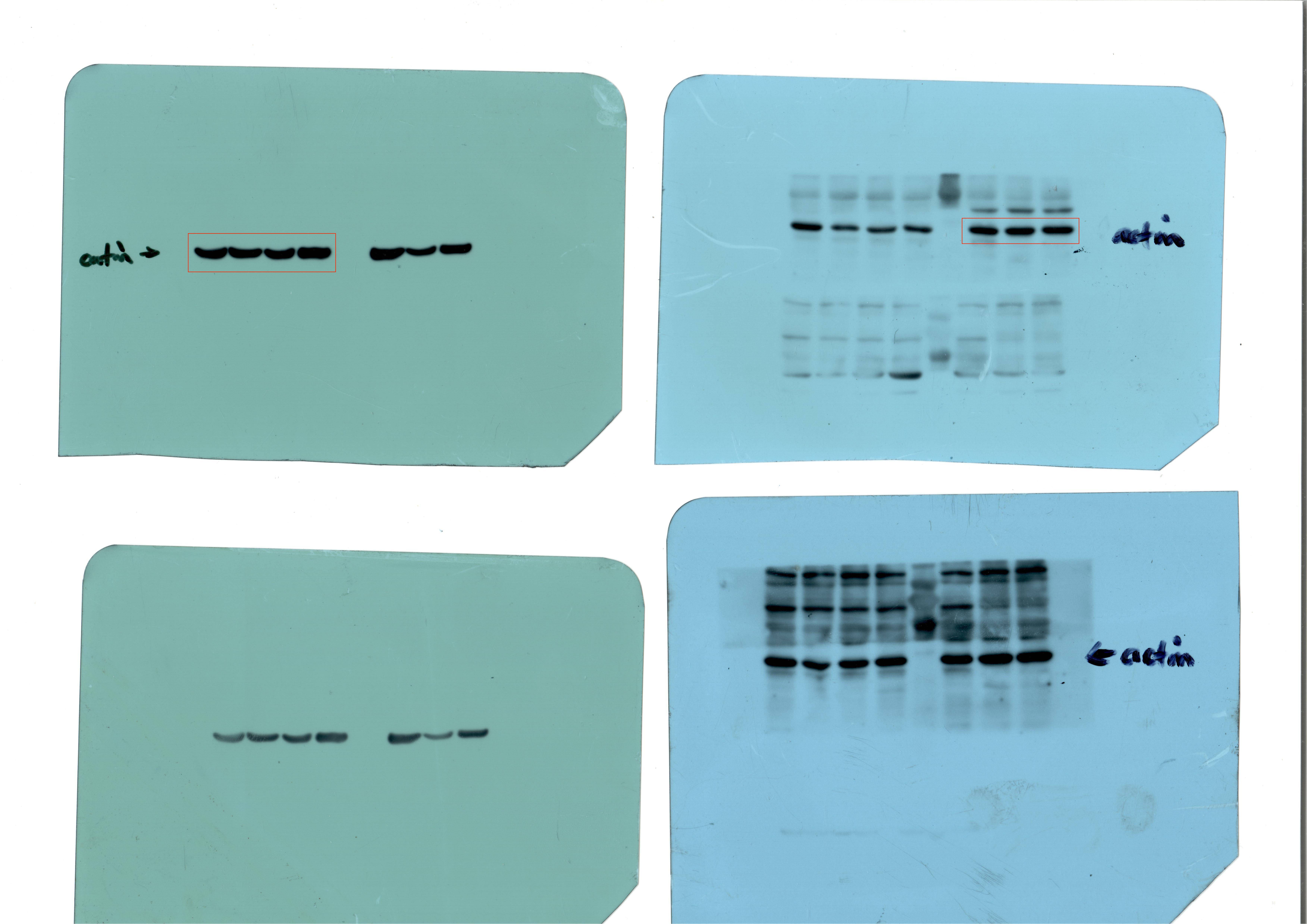

Supplement: Figure 3—source data 1. [file elife-87419-fig3-data1.zip › Figure 3-source data 1/3EF/20201114-WB/Scan3.jpg]

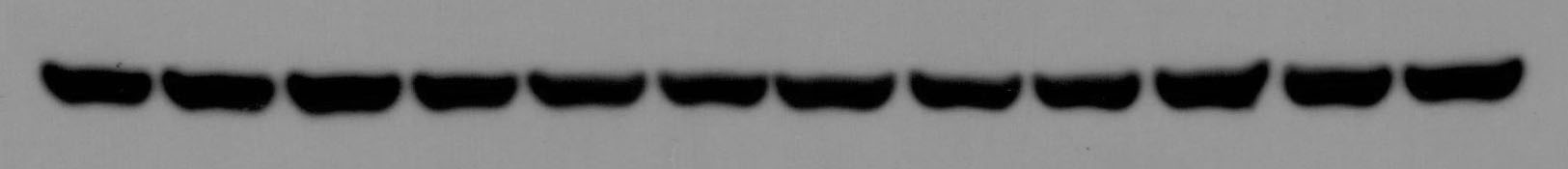

Supplement: Figure 3—figure supplement 1—source data 1. [file elife-87419-fig3-figsupp1-data1.zip › Figure 3-figure supplement 1-source data 1/S3-1BC/20200517-WB/0517-actin.jpg]

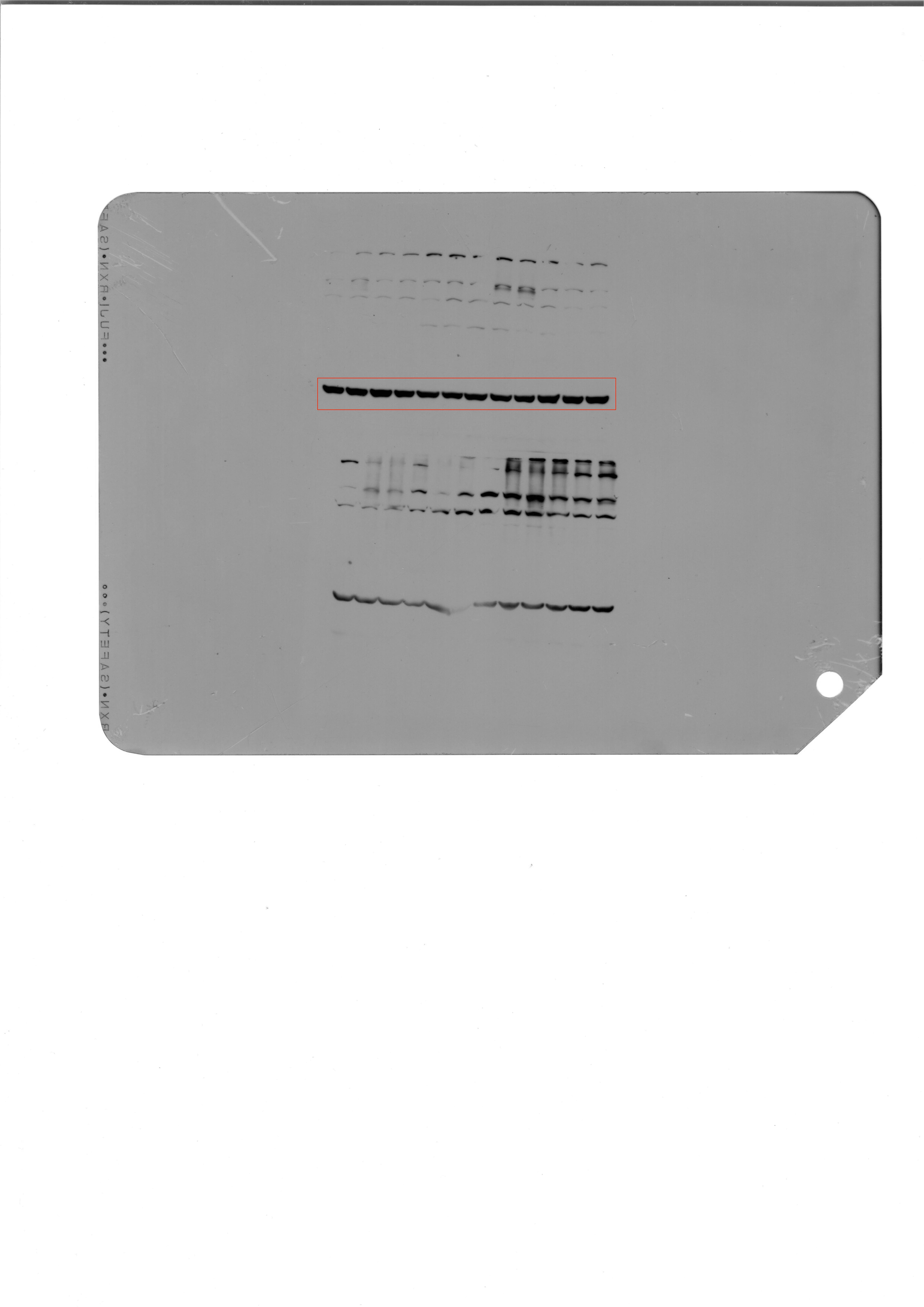

Supplement: Figure 3—figure supplement 1—source data 1. [file elife-87419-fig3-figsupp1-data1.zip › Figure 3-figure supplement 1-source data 1/S3-1BC/20200517-WB/20200517-actin.jpg]

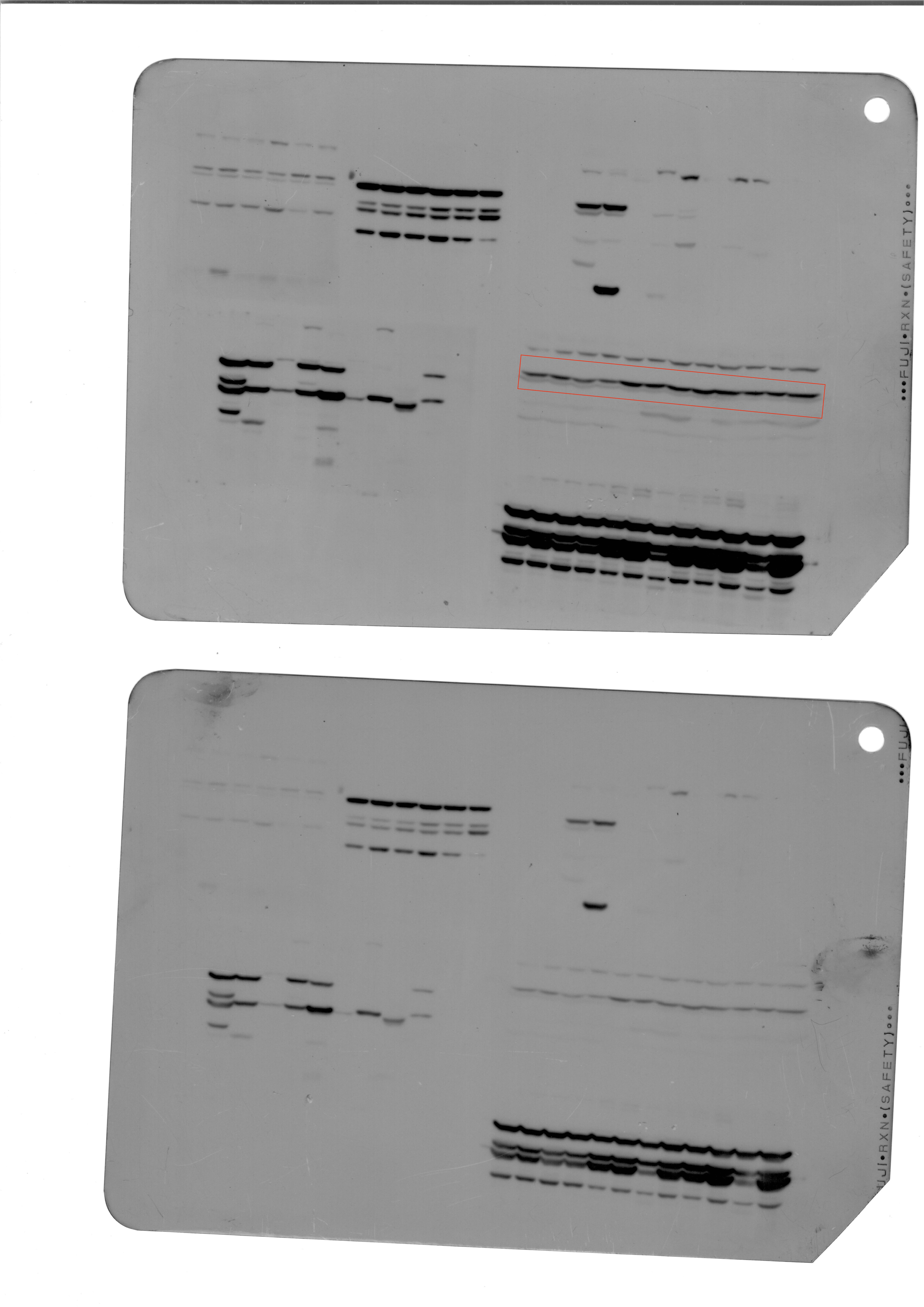

Supplement: Figure 3—figure supplement 1—source data 1. [file elife-87419-fig3-figsupp1-data1.zip › Figure 3-figure supplement 1-source data 1/S3-1BC/20200517-WB/acot12-3.jpg]

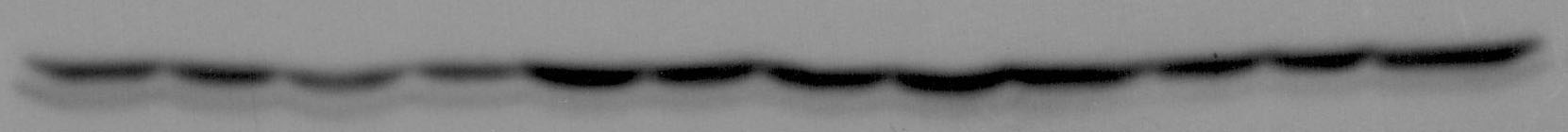

Supplement: Figure 3—figure supplement 1—source data 1. [file elife-87419-fig3-figsupp1-data1.zip › Figure 3-figure supplement 1-source data 1/S3-1BC/20200517-WB/acot12.1.jpg]

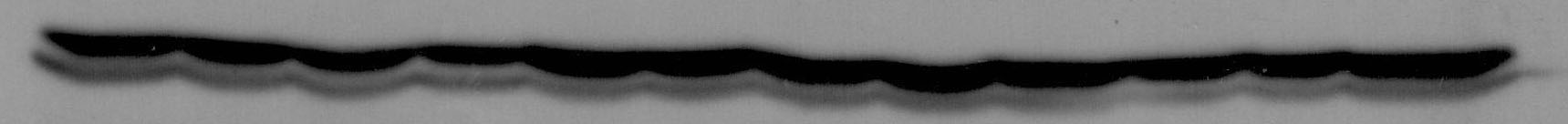

Supplement: Figure 3—figure supplement 1—source data 1. [file elife-87419-fig3-figsupp1-data1.zip › Figure 3-figure supplement 1-source data 1/S3-1BC/20200517-WB/acot12.jpg]

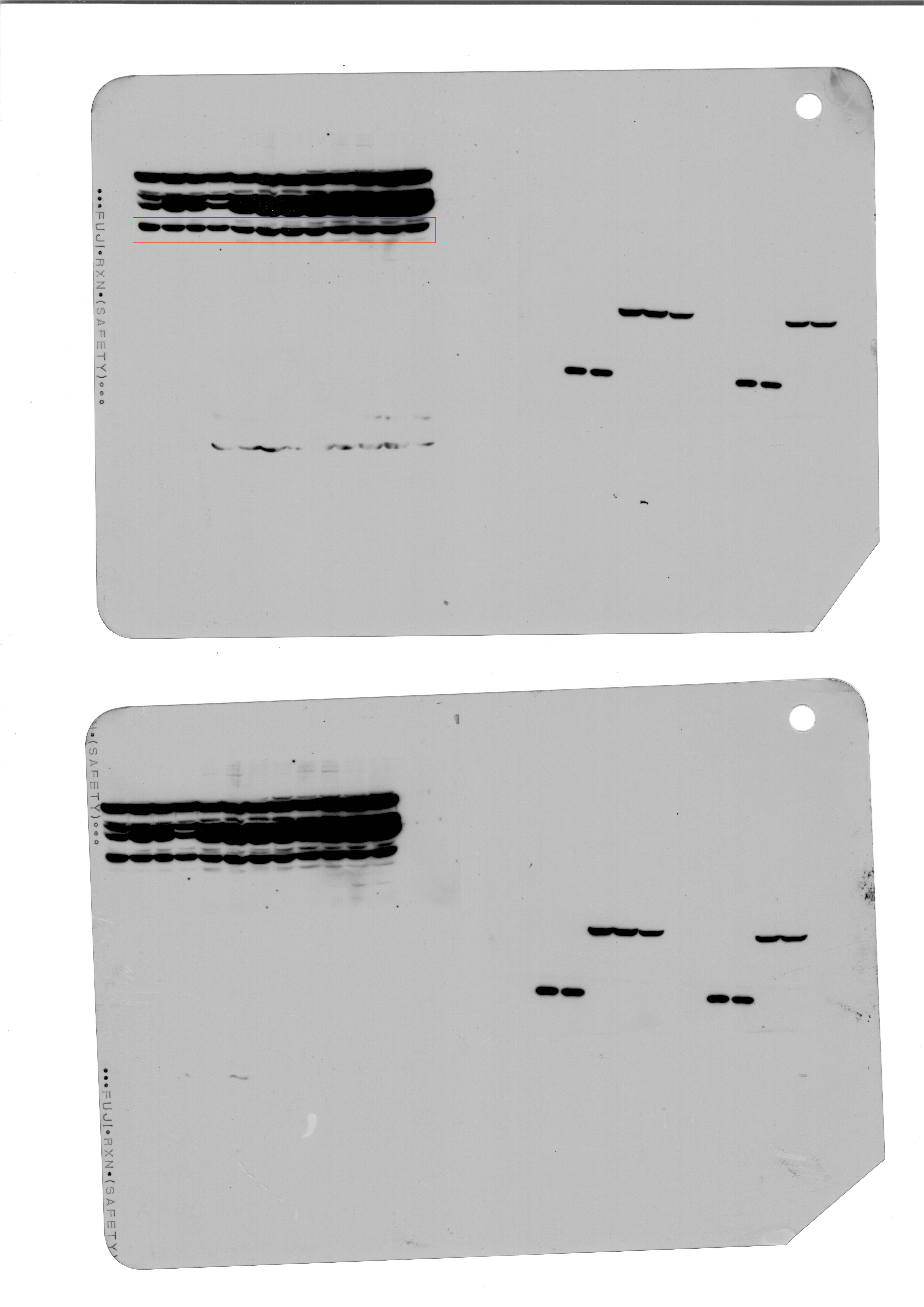

Supplement: Figure 3—figure supplement 1—source data 1. [file elife-87419-fig3-figsupp1-data1.zip › Figure 3-figure supplement 1-source data 1/S3-1BC/20200517-WB/acot8-2.jpg]

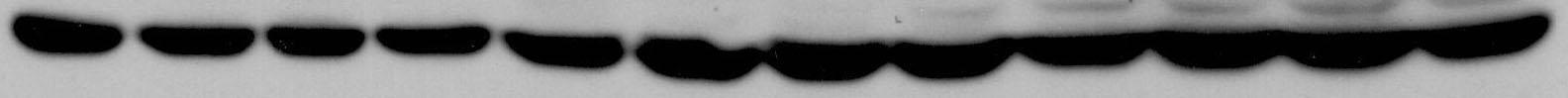

Supplement: Figure 3—figure supplement 1—source data 1. [file elife-87419-fig3-figsupp1-data1.zip › Figure 3-figure supplement 1-source data 1/S3-1BC/20200517-WB/acot8.1.jpg]

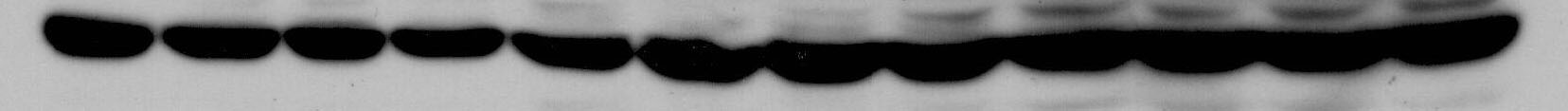

Supplement: Figure 3—figure supplement 1—source data 1. [file elife-87419-fig3-figsupp1-data1.zip › Figure 3-figure supplement 1-source data 1/S3-1BC/20200517-WB/acot8.jpg]

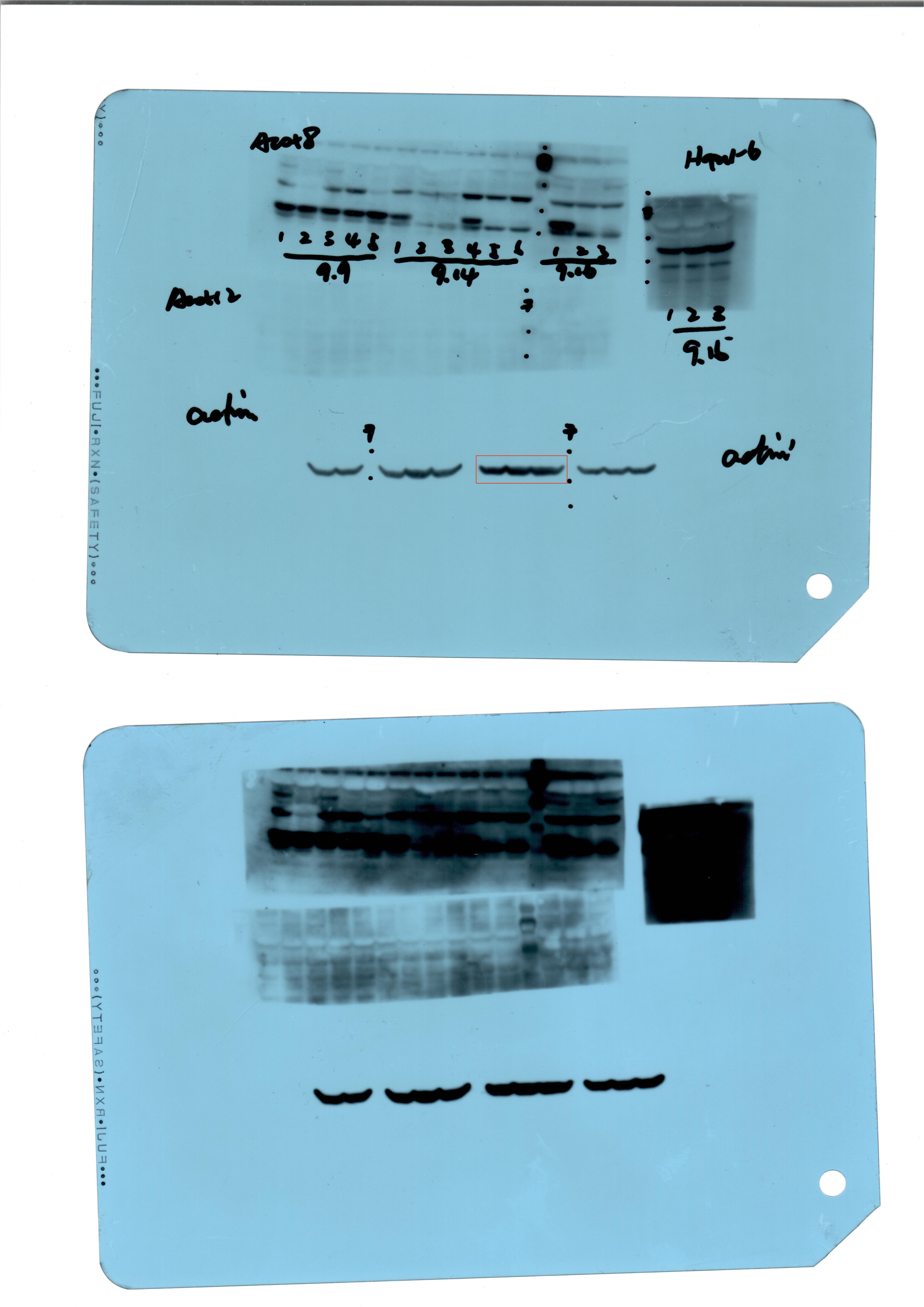

Supplement: Figure 3—figure supplement 1—source data 1. [file elife-87419-fig3-figsupp1-data1.zip › Figure 3-figure supplement 1-source data 1/S3-1G/20200915-WB/1.jpg]

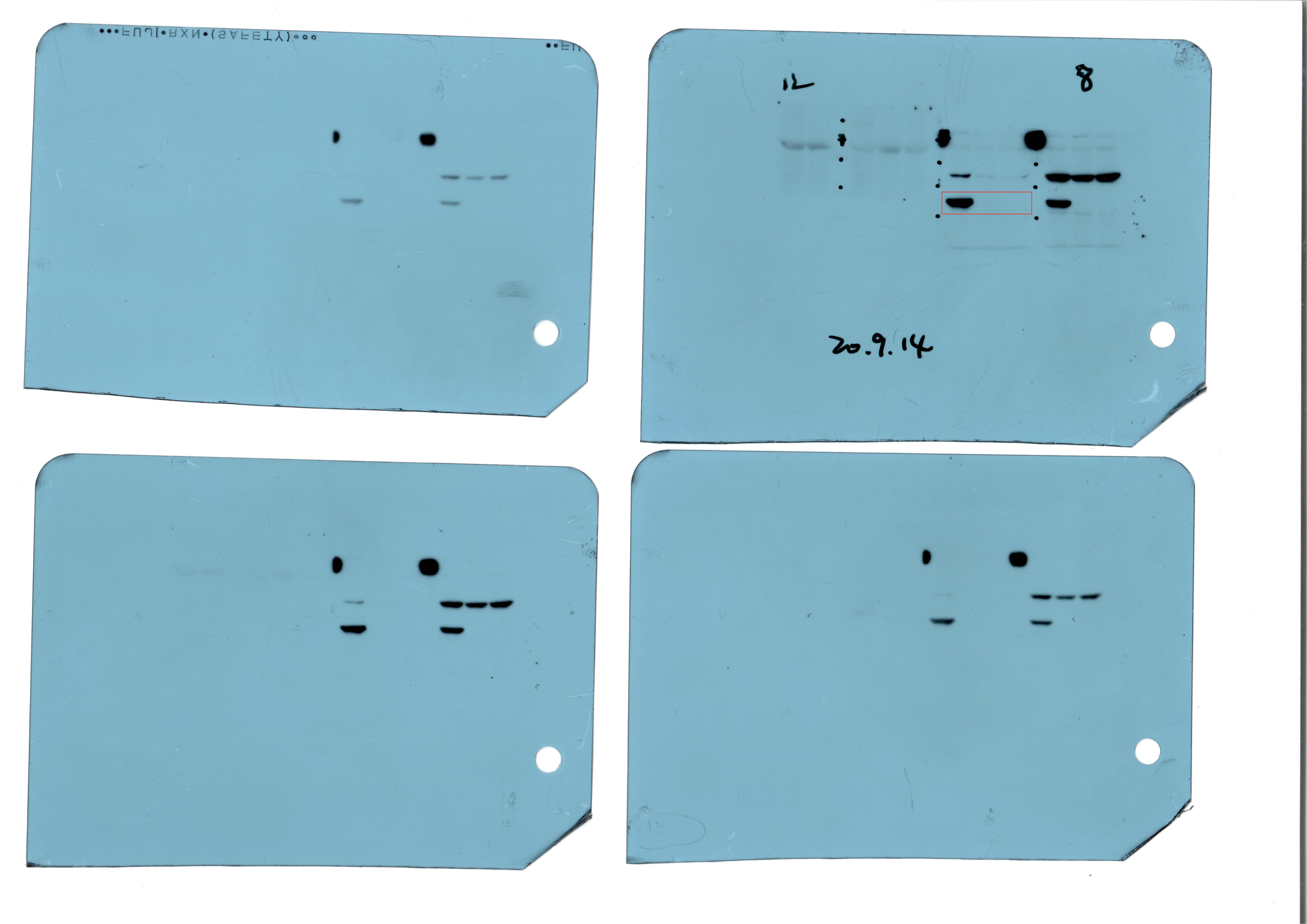

Supplement: Figure 3—figure supplement 1—source data 1. [file elife-87419-fig3-figsupp1-data1.zip › Figure 3-figure supplement 1-source data 1/S3-1G/20200915-WB/2.jpg]

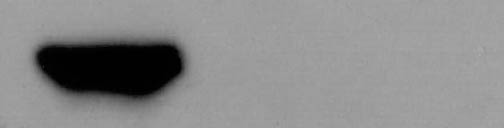

Supplement: Figure 3—figure supplement 1—source data 1. [file elife-87419-fig3-figsupp1-data1.zip › Figure 3-figure supplement 1-source data 1/S3-1G/20200915-WB/acot8-1.jpg]

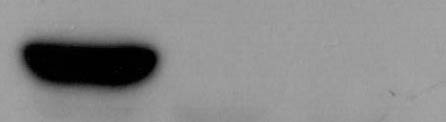

Supplement: Figure 3—figure supplement 1—source data 1. [file elife-87419-fig3-figsupp1-data1.zip › Figure 3-figure supplement 1-source data 1/S3-1G/20200915-WB/acot8-2.jpg]

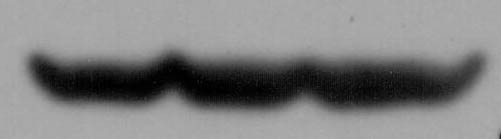

Supplement: Figure 3—figure supplement 1—source data 1. [file elife-87419-fig3-figsupp1-data1.zip › Figure 3-figure supplement 1-source data 1/S3-1G/20200915-WB/actin1.jpg]

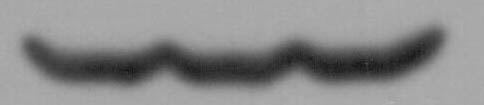

Supplement: Figure 3—figure supplement 1—source data 1. [file elife-87419-fig3-figsupp1-data1.zip › Figure 3-figure supplement 1-source data 1/S3-1G/20200915-WB/actin2.jpg]

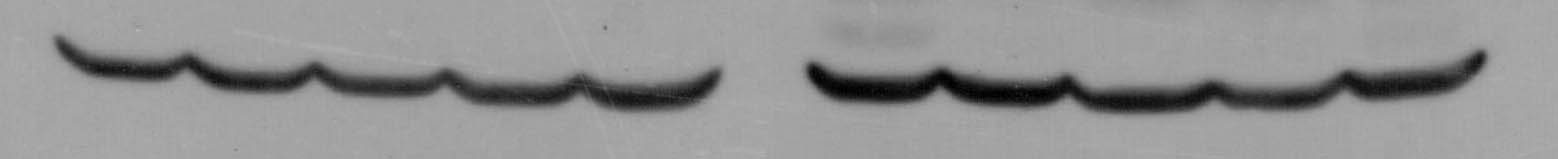

Supplement: Figure 4—source data 1. [file elife-87419-fig4-data1.zip › Figure 4-source data 1/4A-4D/20210414-WB/12-actin.jpg]

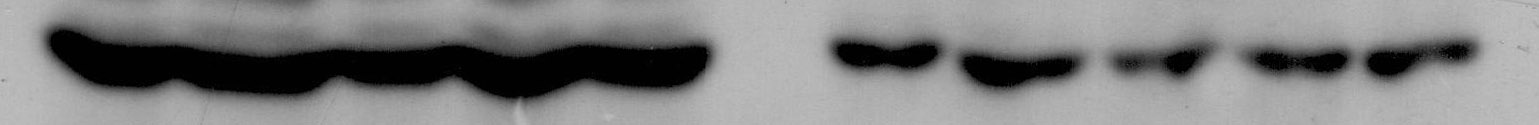

Supplement: Figure 4—source data 1. [file elife-87419-fig4-data1.zip › Figure 4-source data 1/4A-4D/20210414-WB/1n-8.jpg]

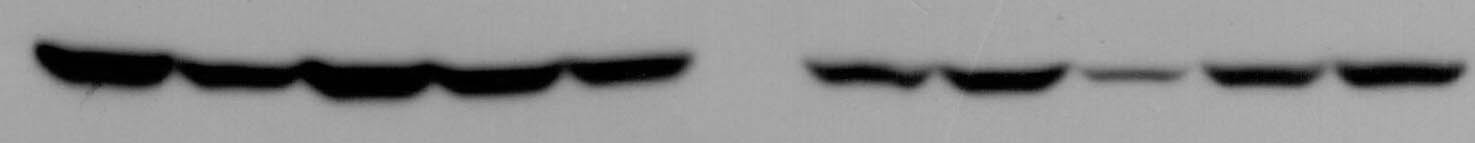

Supplement: Figure 4—source data 1. [file elife-87419-fig4-data1.zip › Figure 4-source data 1/4A-4D/20210414-WB/2-12.jpg]

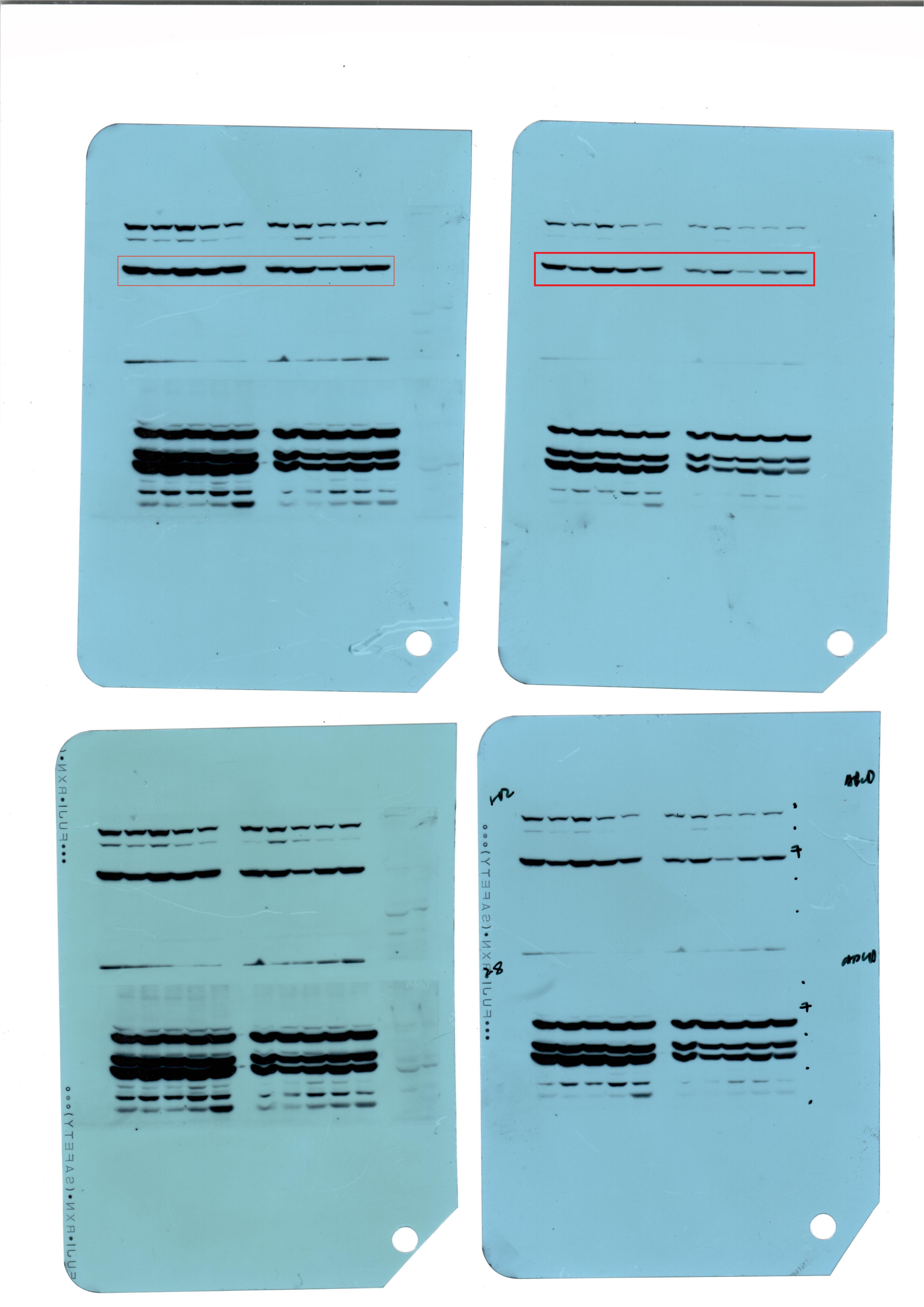

Supplement: Figure 4—source data 1. [file elife-87419-fig4-data1.zip › Figure 4-source data 1/4A-4D/20210414-WB/2.jpg]

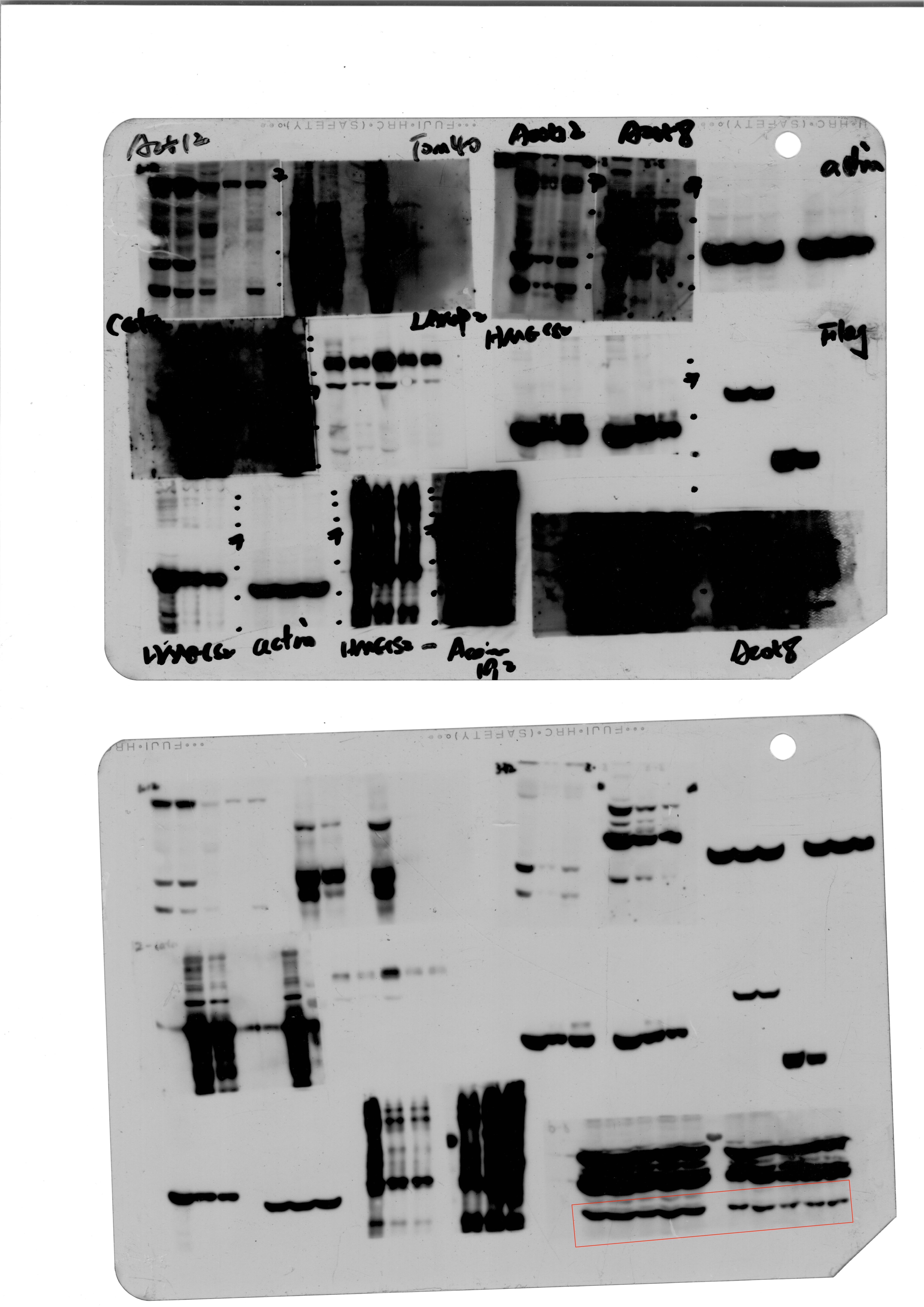

Supplement: Figure 4—source data 1. [file elife-87419-fig4-data1.zip › Figure 4-source data 1/4A-4D/20210414-WB/8-1.jpg]

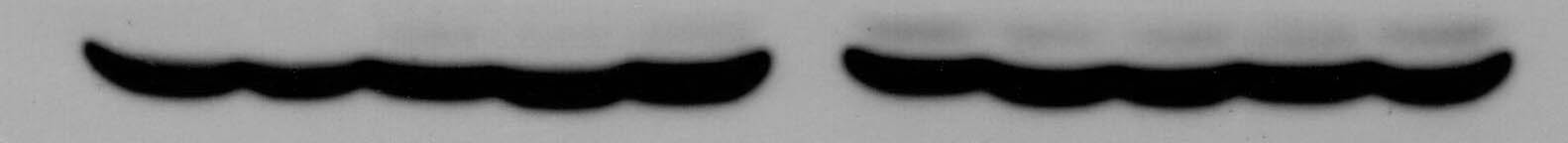

Supplement: Figure 4—source data 1. [file elife-87419-fig4-data1.zip › Figure 4-source data 1/4A-4D/20210414-WB/8-actin.jpg]

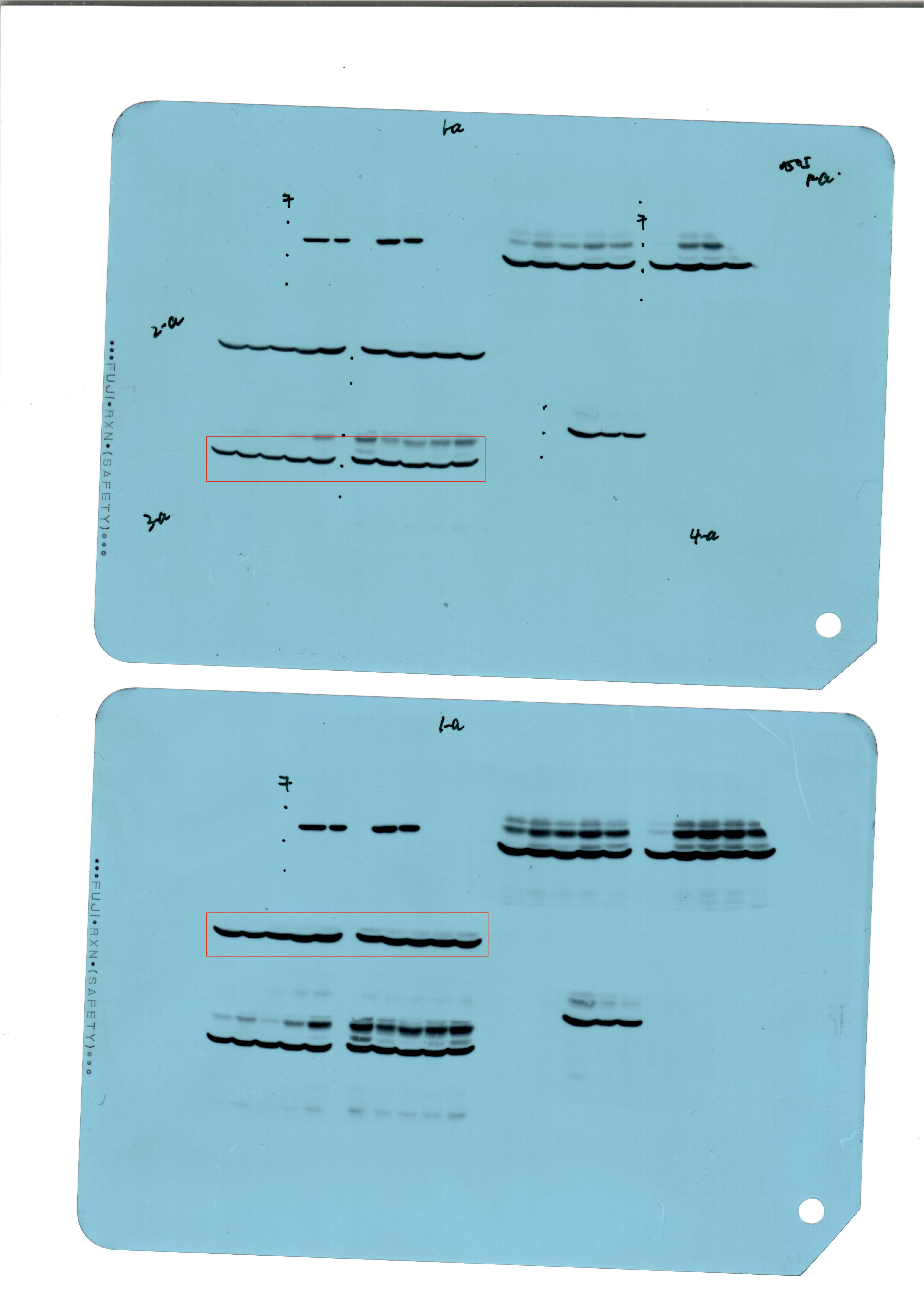

Supplement: Figure 4—source data 1. [file elife-87419-fig4-data1.zip › Figure 4-source data 1/4A-4D/20210414-WB/Scan3.jpg]

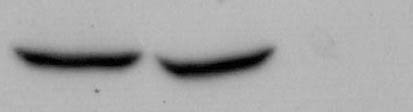

Supplement: Figure 4—source data 1. [file elife-87419-fig4-data1.zip › Figure 4-source data 1/4G4H/20230130-WB/扫描01-12.jpg]

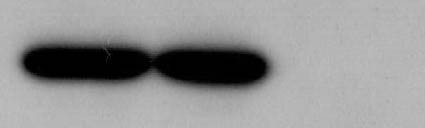

Supplement: Figure 4—source data 1. [file elife-87419-fig4-data1.zip › Figure 4-source data 1/4G4H/20230130-WB/扫描01-8.jpg]

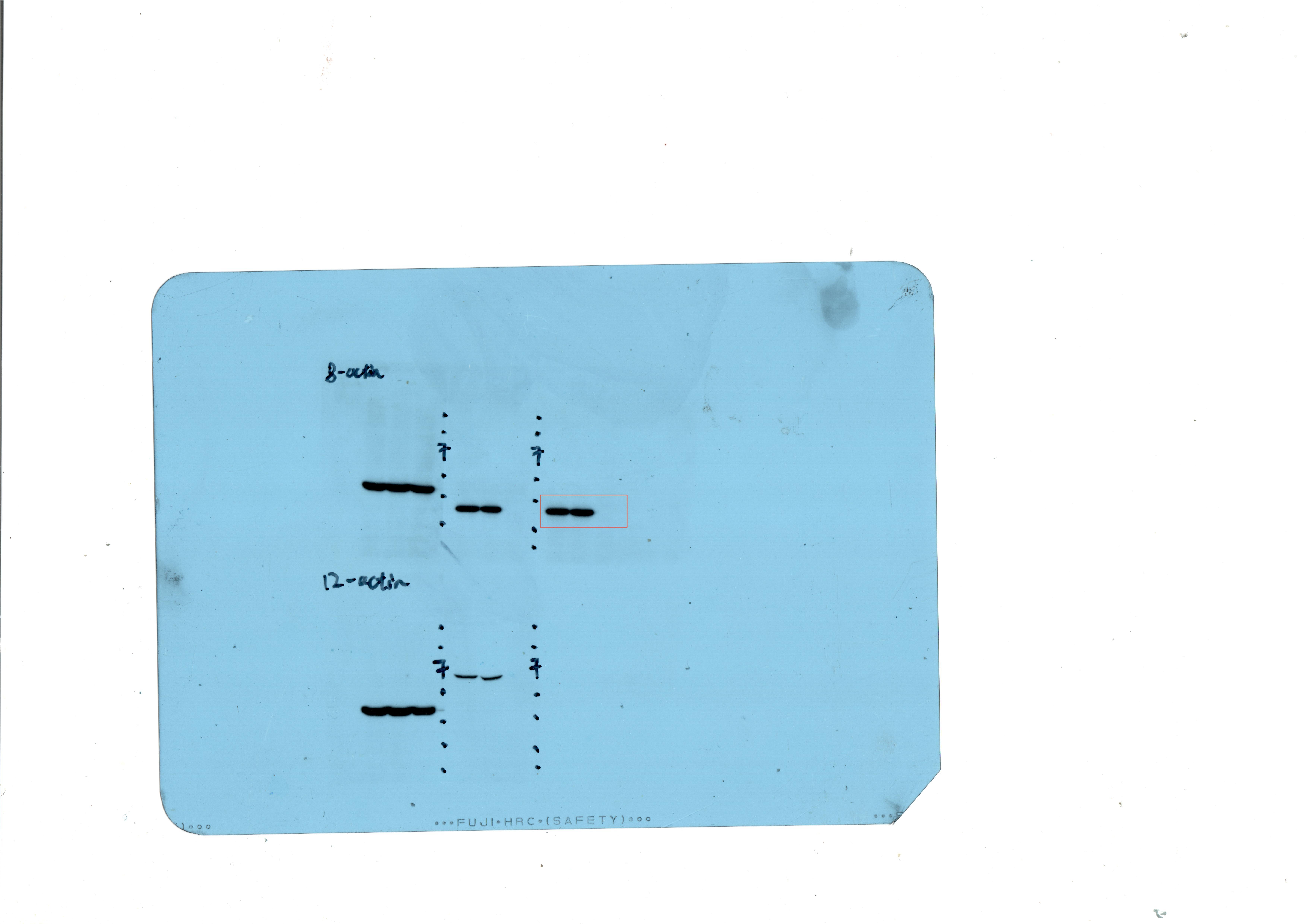

Supplement: Figure 4—source data 1. [file elife-87419-fig4-data1.zip › Figure 4-source data 1/4G4H/20230130-WB/扫描01.jpg]

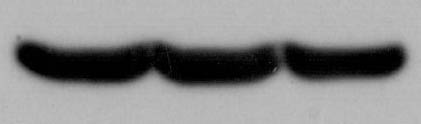

Supplement: Figure 4—source data 1. [file elife-87419-fig4-data1.zip › Figure 4-source data 1/4G4H/20230130-WB/扫描02-12-actin.jpg]

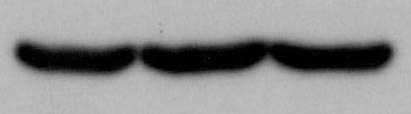

Supplement: Figure 4—source data 1. [file elife-87419-fig4-data1.zip › Figure 4-source data 1/4G4H/20230130-WB/扫描02-8-actin.jpg]

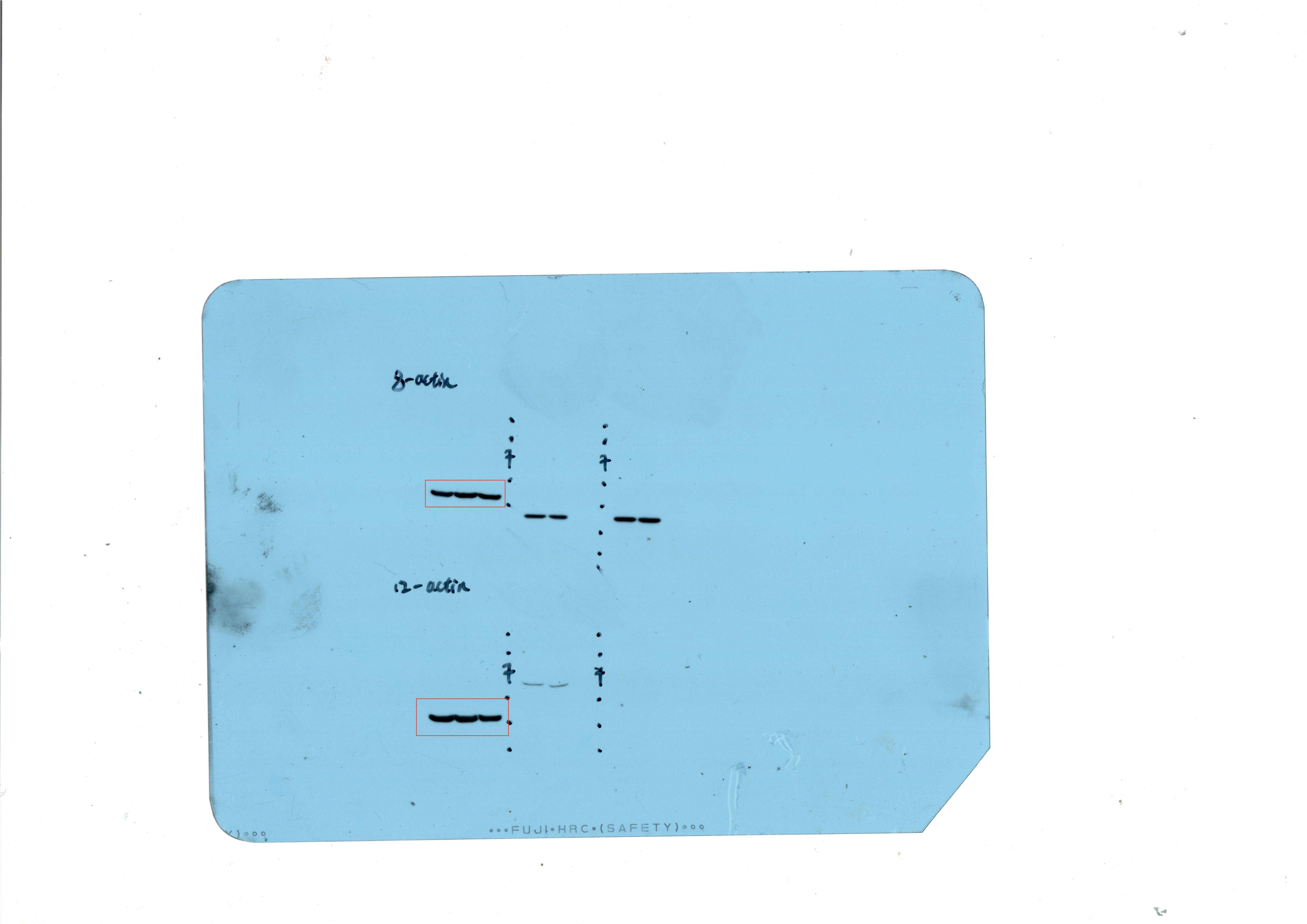

Supplement: Figure 4—source data 1. [file elife-87419-fig4-data1.zip › Figure 4-source data 1/4G4H/20230130-WB/扫描02.jpg]

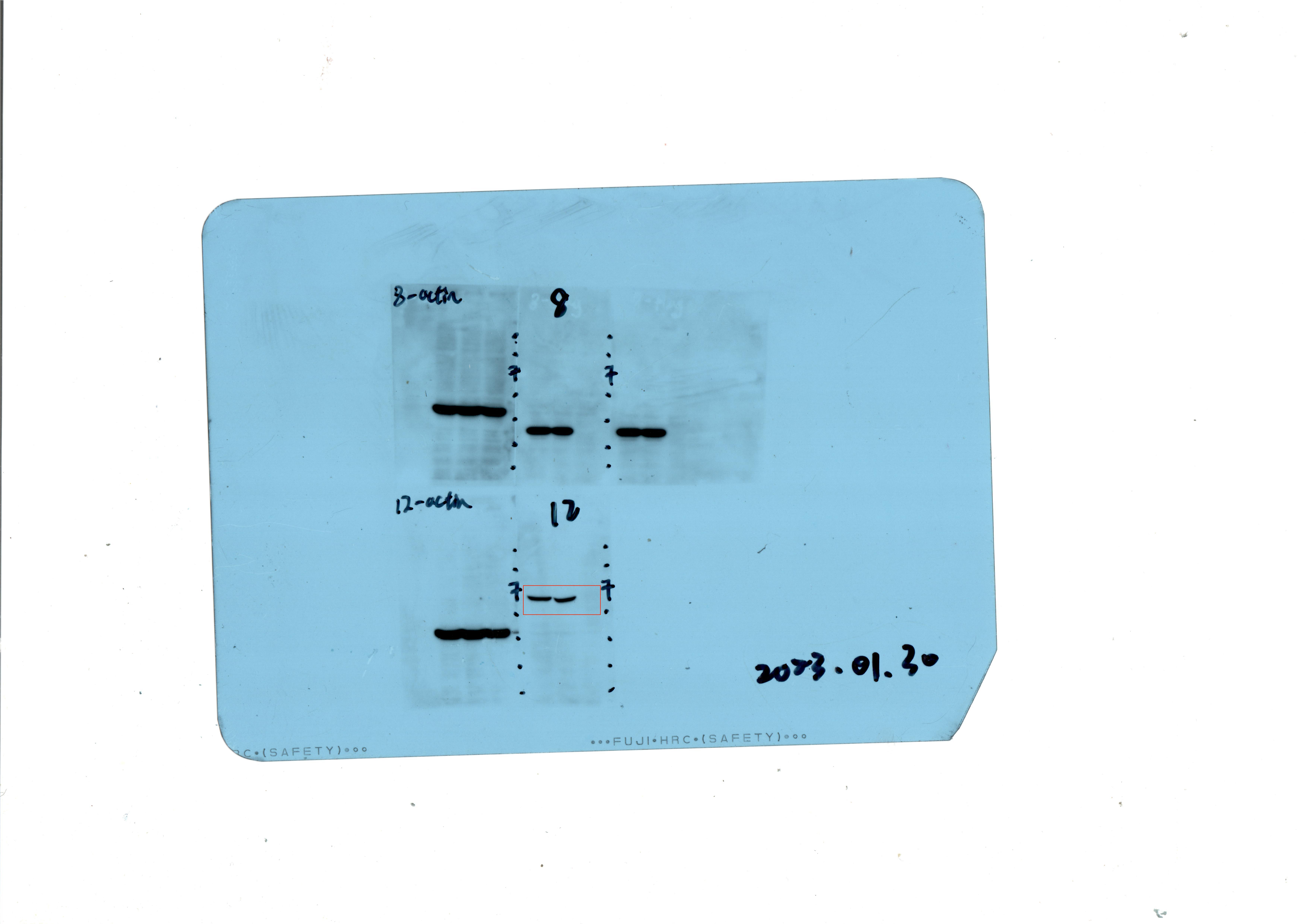

Supplement: Figure 4—source data 1. [file elife-87419-fig4-data1.zip › Figure 4-source data 1/4G4H/20230130-WB/扫描03.jpg]

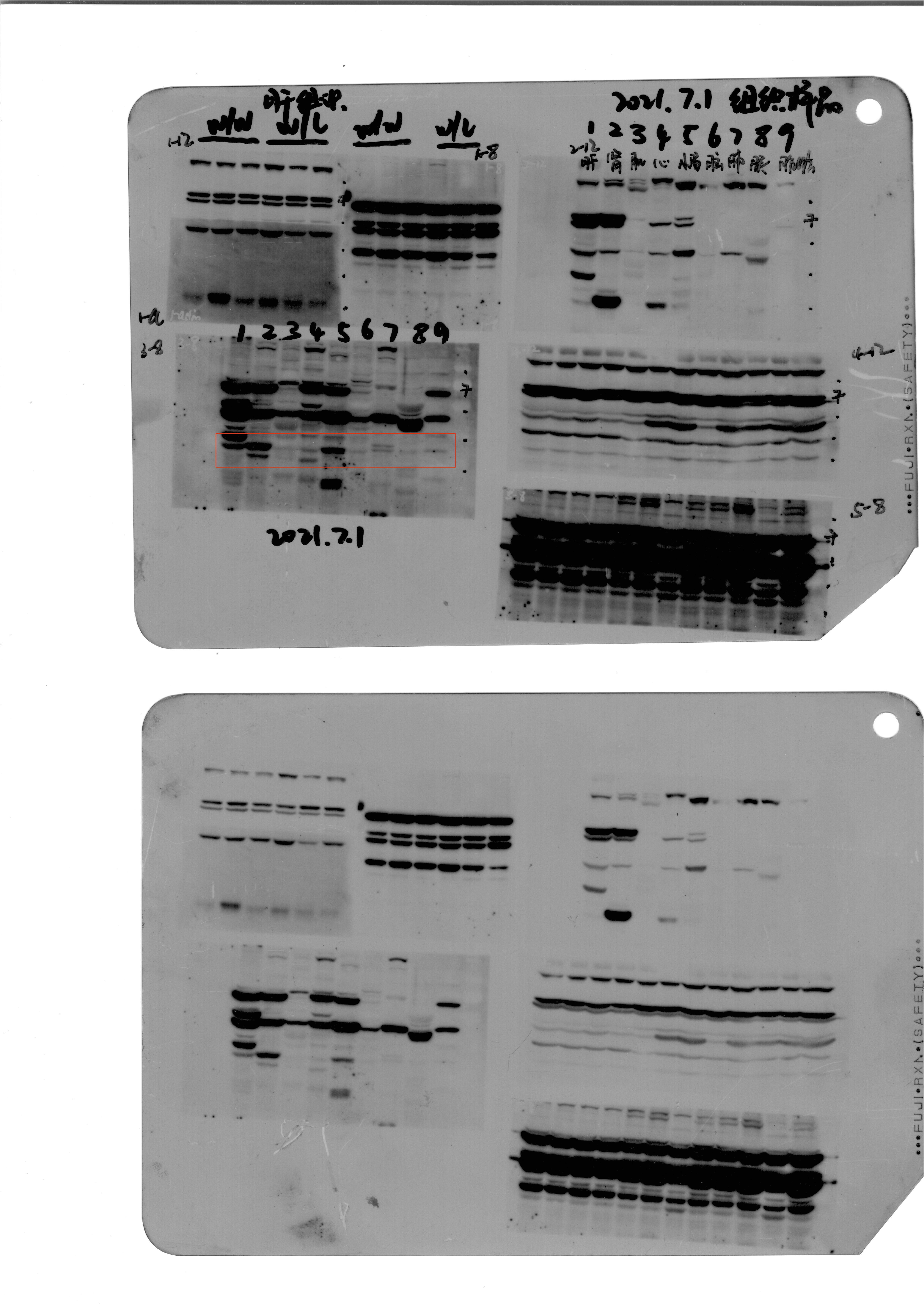

Supplement: Figure 4—figure supplement 2—source data 1. [file elife-87419-fig4-figsupp2-data1.zip › Figure 4-figure supplement 2-source data 1/S4-2B/20210701-WB/1.jpg]

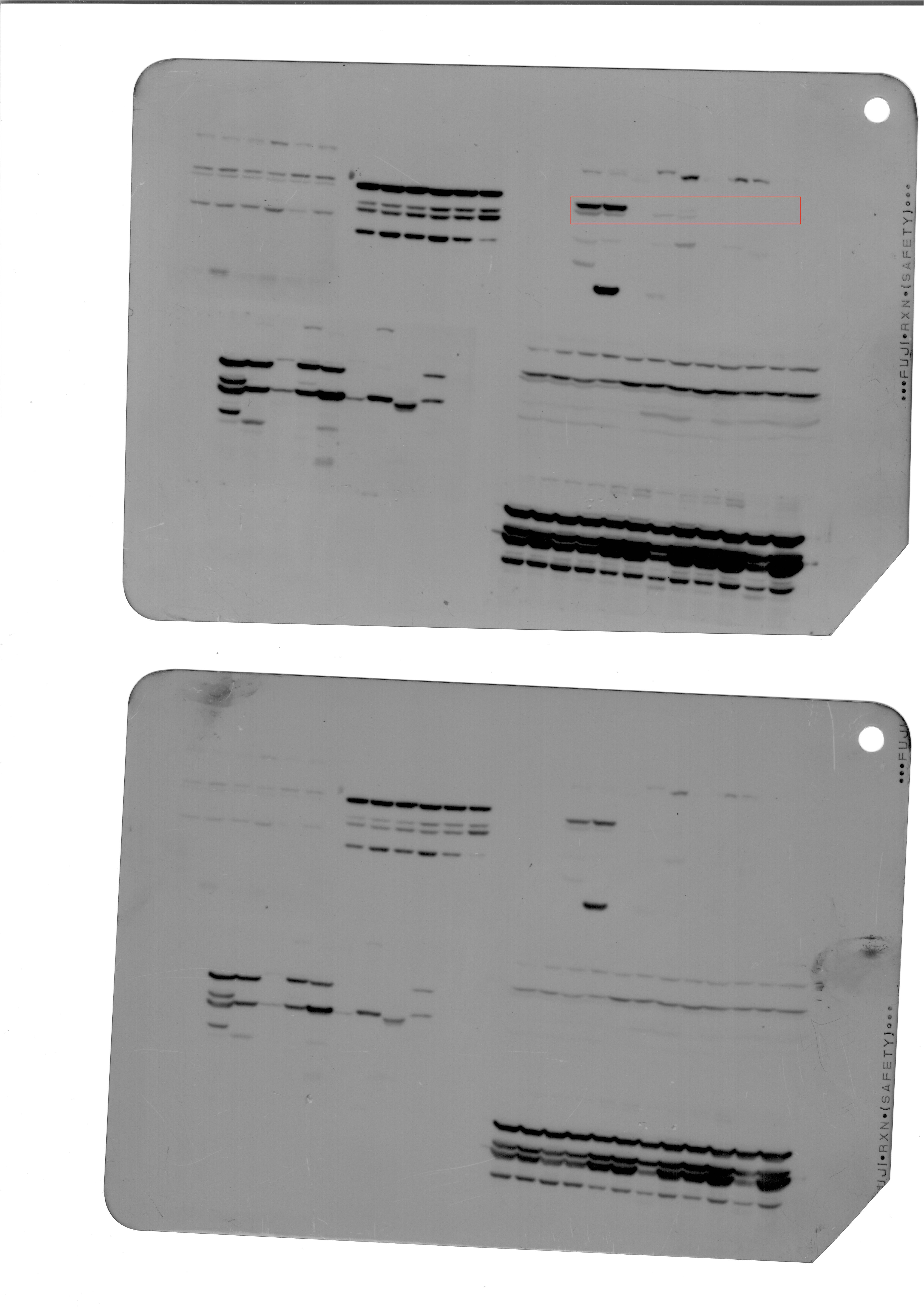

Supplement: Figure 4—figure supplement 2—source data 1. [file elife-87419-fig4-figsupp2-data1.zip › Figure 4-figure supplement 2-source data 1/S4-2B/20210701-WB/2.jpg]

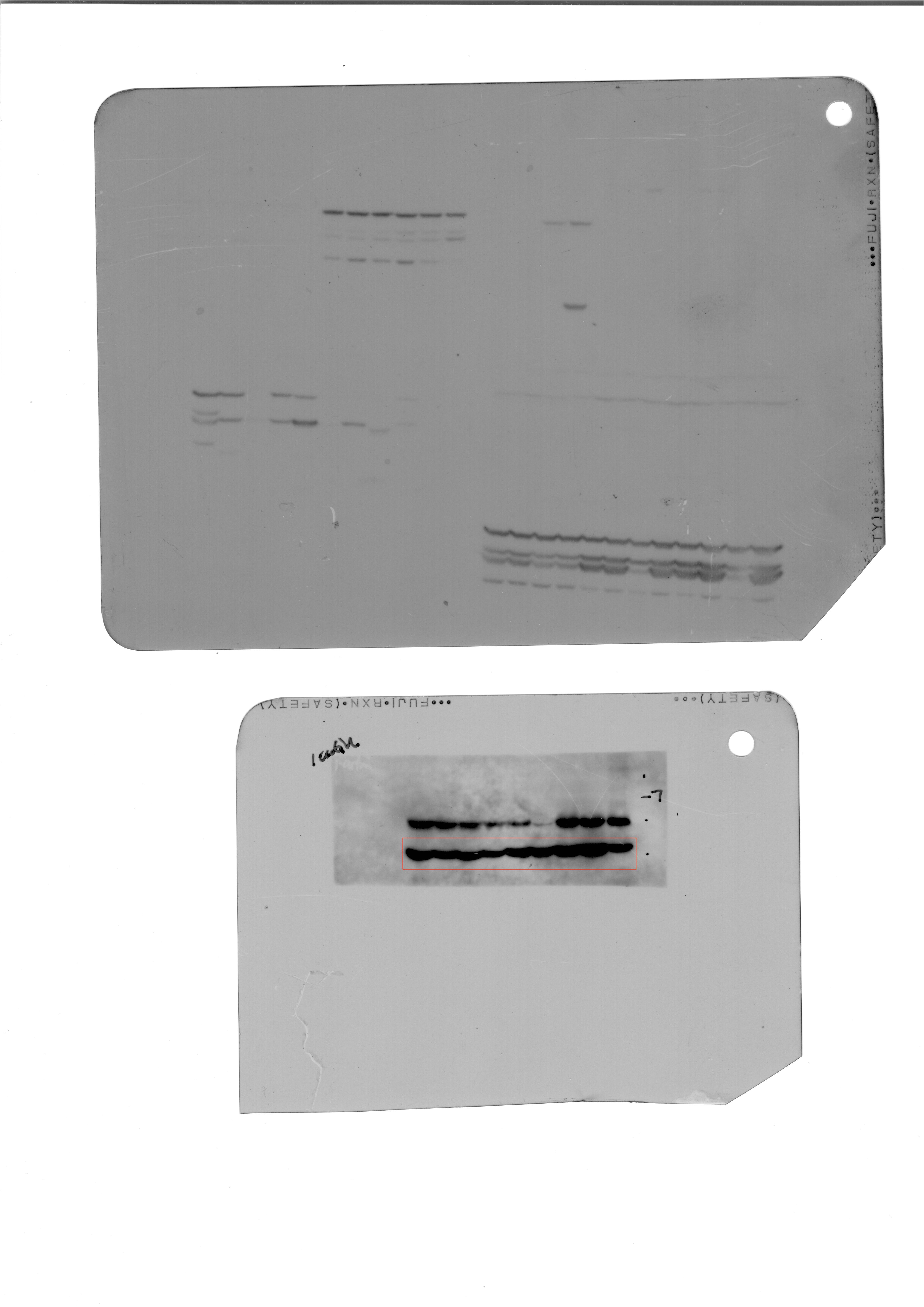

Supplement: Figure 4—figure supplement 2—source data 1. [file elife-87419-fig4-figsupp2-data1.zip › Figure 4-figure supplement 2-source data 1/S4-2B/20210701-WB/3.jpg]

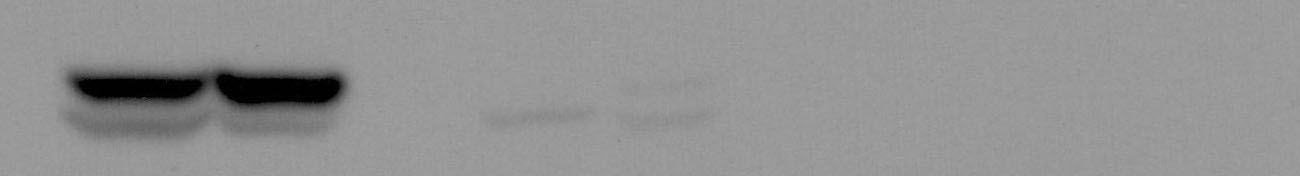

Supplement: Figure 4—figure supplement 2—source data 1. [file elife-87419-fig4-figsupp2-data1.zip › Figure 4-figure supplement 2-source data 1/S4-2B/20210701-WB/acot12.jpg]

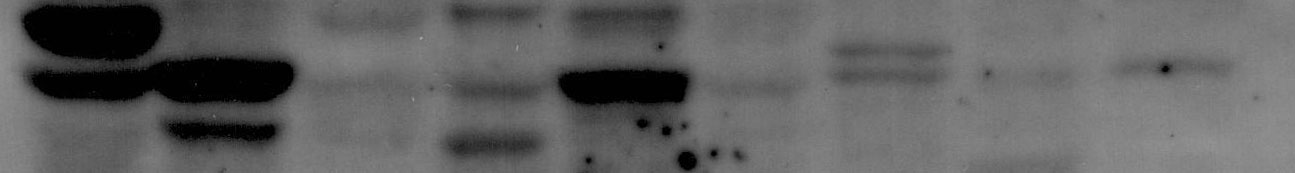

Supplement: Figure 4—figure supplement 2—source data 1. [file elife-87419-fig4-figsupp2-data1.zip › Figure 4-figure supplement 2-source data 1/S4-2B/20210701-WB/ACOT8.jpg]

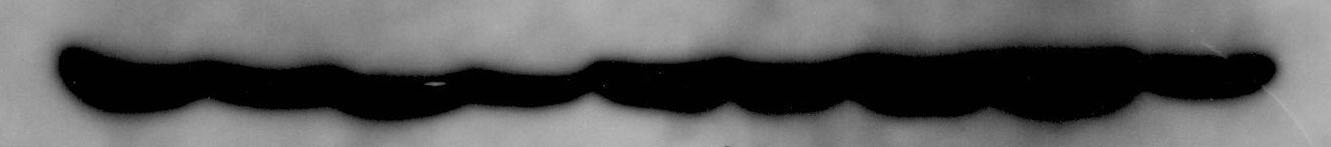

Supplement: Figure 4—figure supplement 2—source data 1. [file elife-87419-fig4-figsupp2-data1.zip › Figure 4-figure supplement 2-source data 1/S4-2B/20210701-WB/actin.jpg]

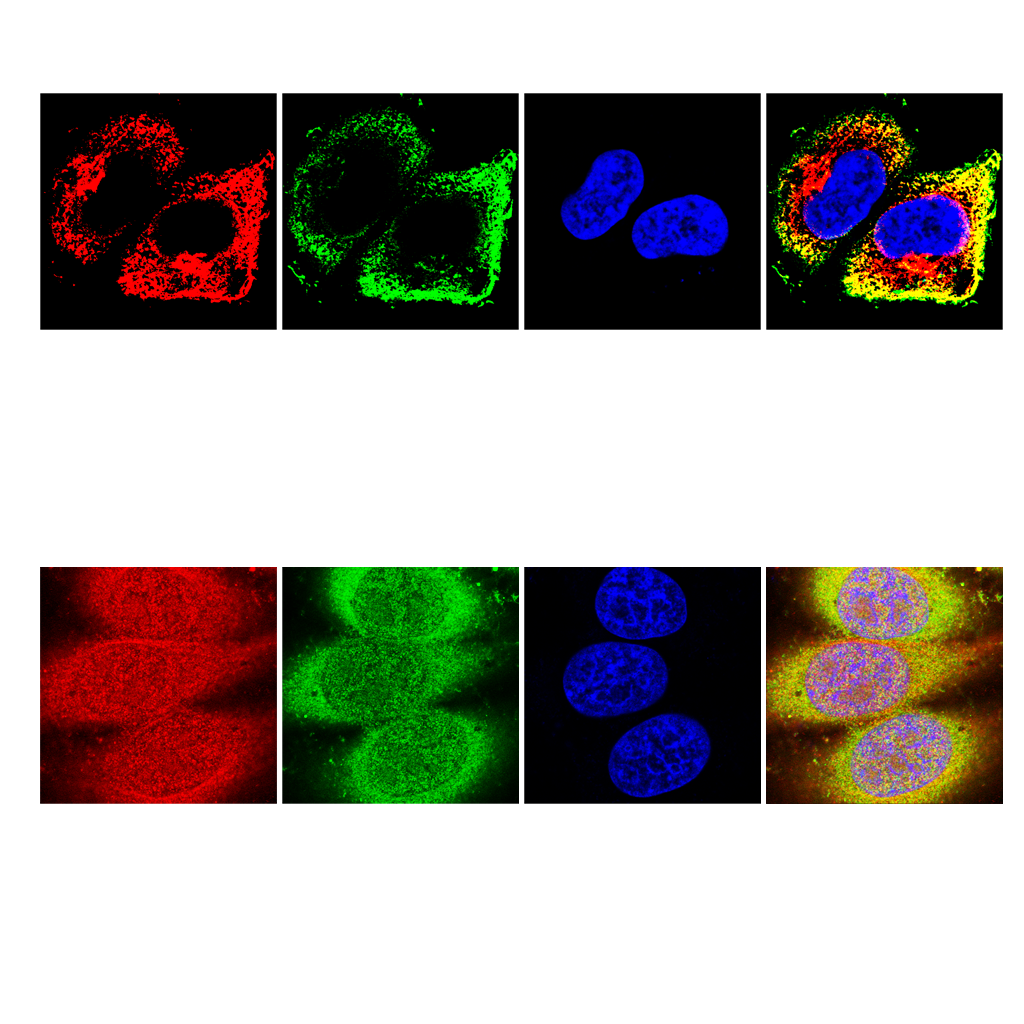

Supplement: Figure 5—source data 1. [file elife-87419-fig5-data1.zip › Figure 5-source data 1/5A/20210806-0907-1123-staining/1.tif]

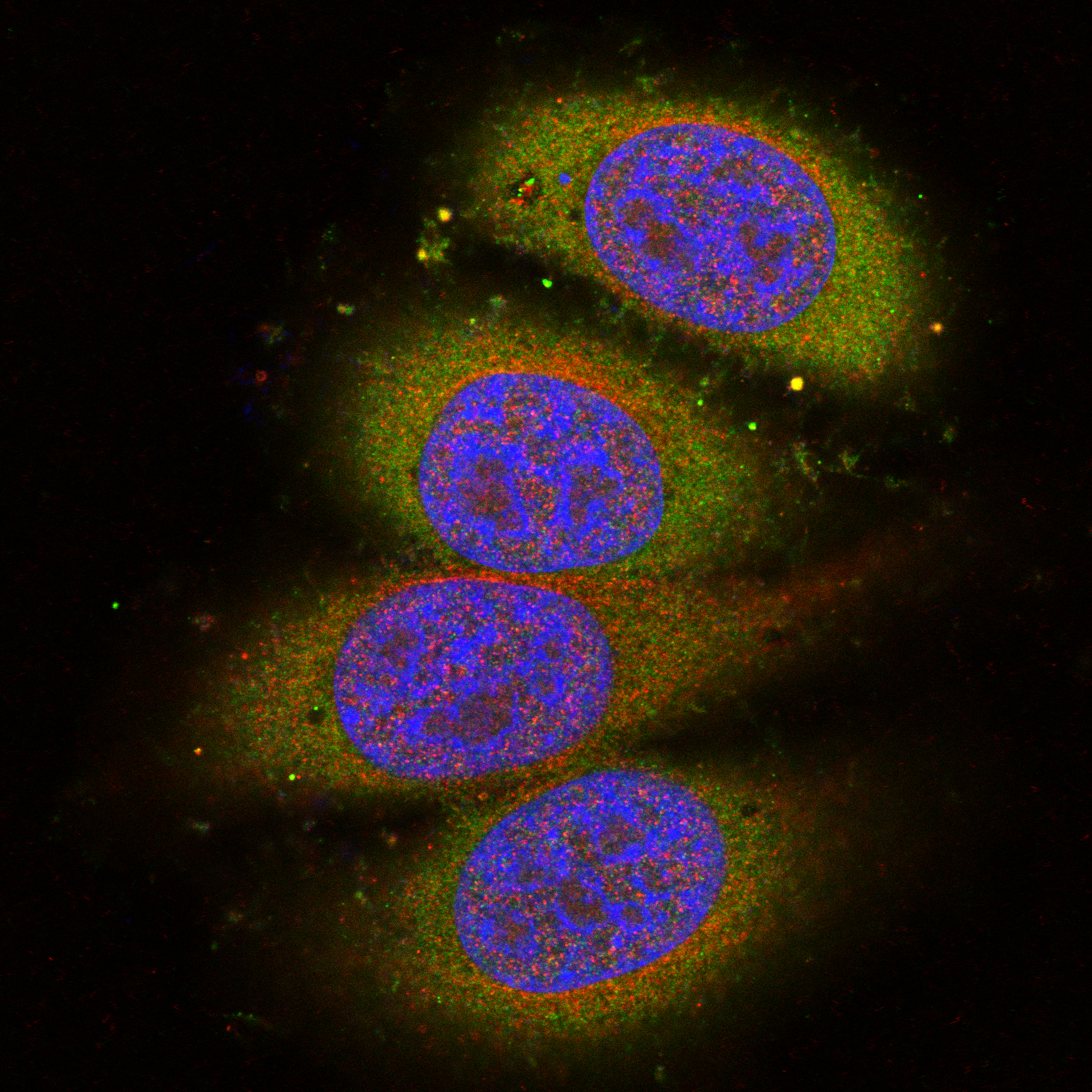

Supplement: Figure 5—source data 1. [file elife-87419-fig5-data1.zip › Figure 5-source data 1/5A/20210806-0907-1123-staining/20210907-15_Series001.tif]

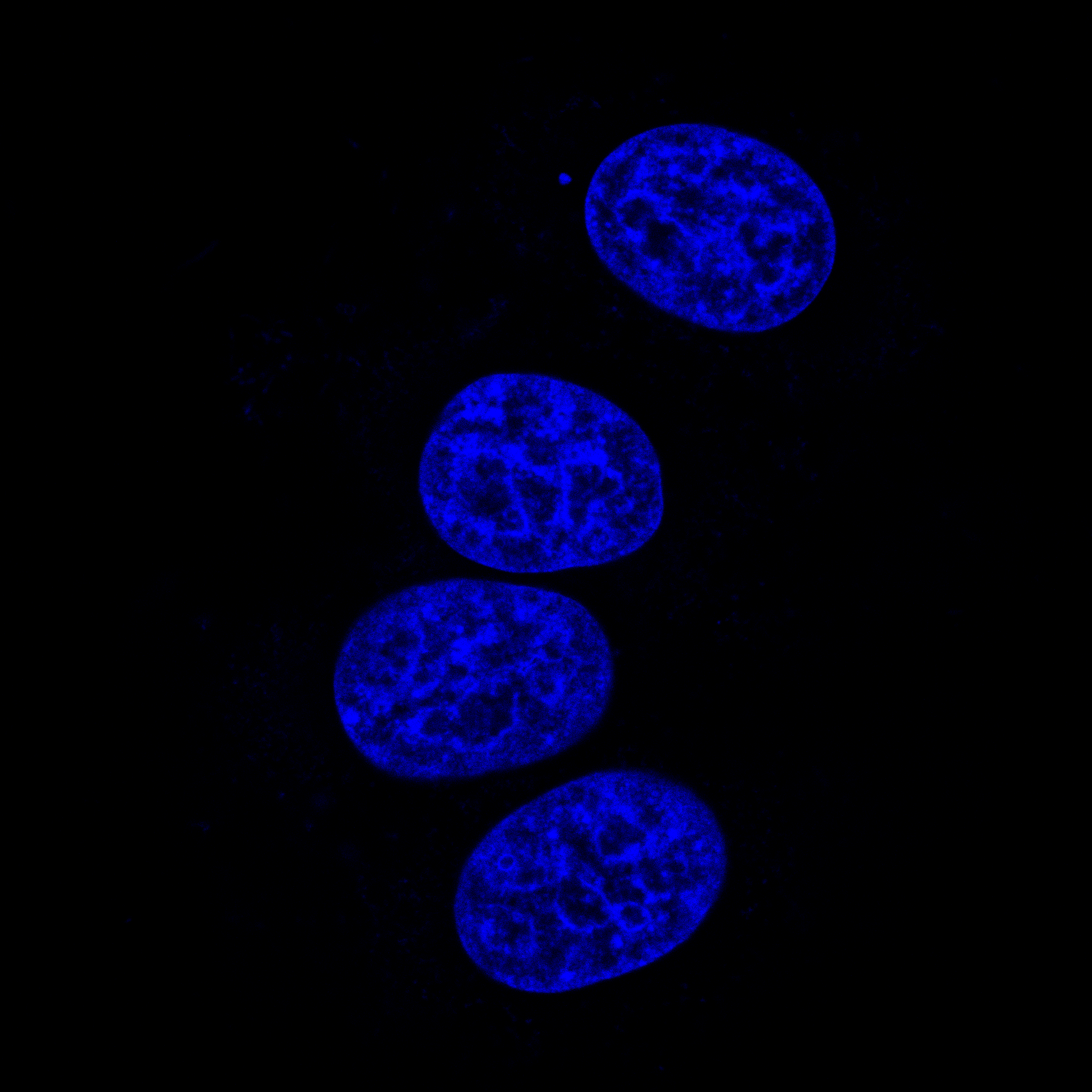

Supplement: Figure 5—source data 1. [file elife-87419-fig5-data1.zip › Figure 5-source data 1/5A/20210806-0907-1123-staining/20210907-15_Series001_ch00.tif]

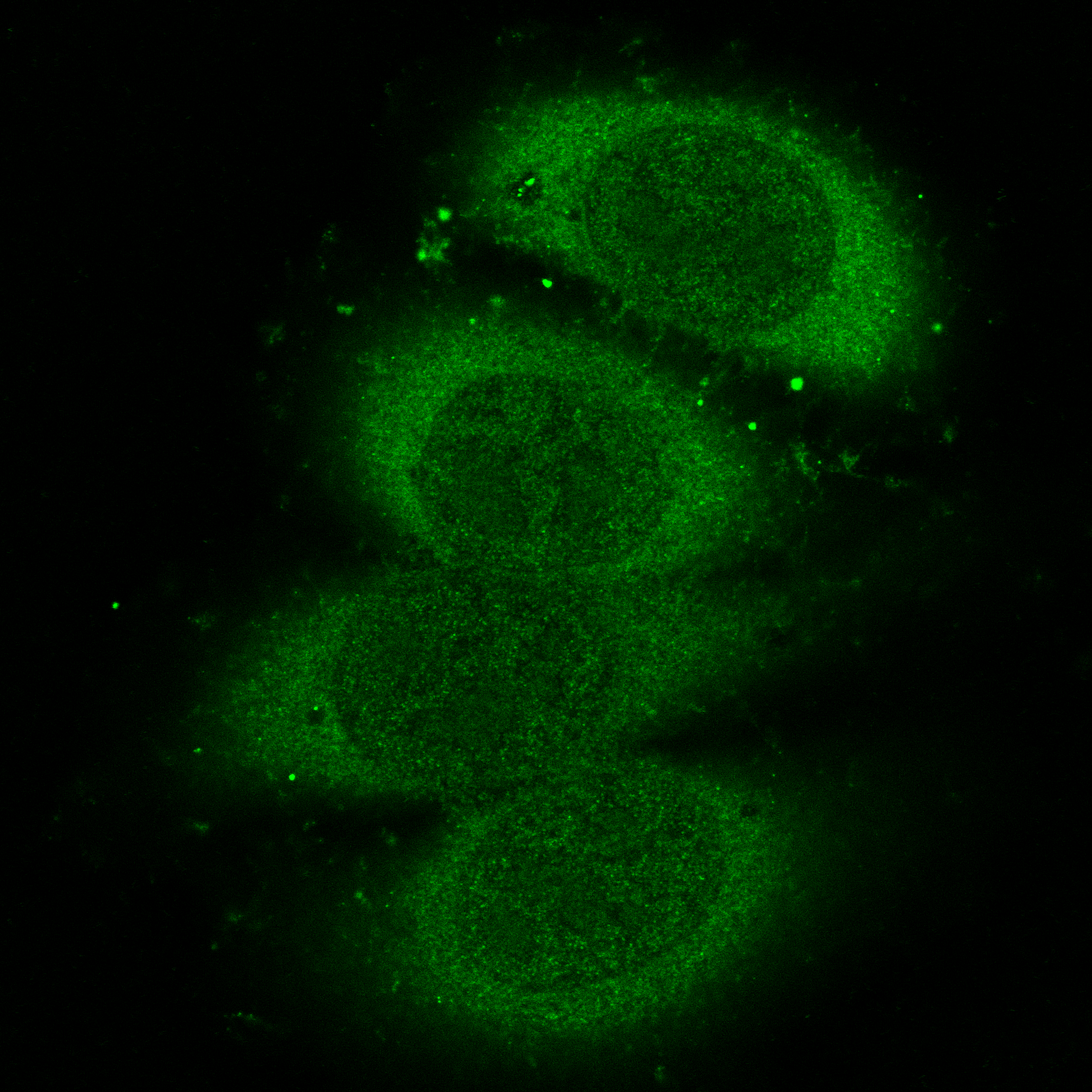

Supplement: Figure 5—source data 1. [file elife-87419-fig5-data1.zip › Figure 5-source data 1/5A/20210806-0907-1123-staining/20210907-15_Series001_ch01.tif]

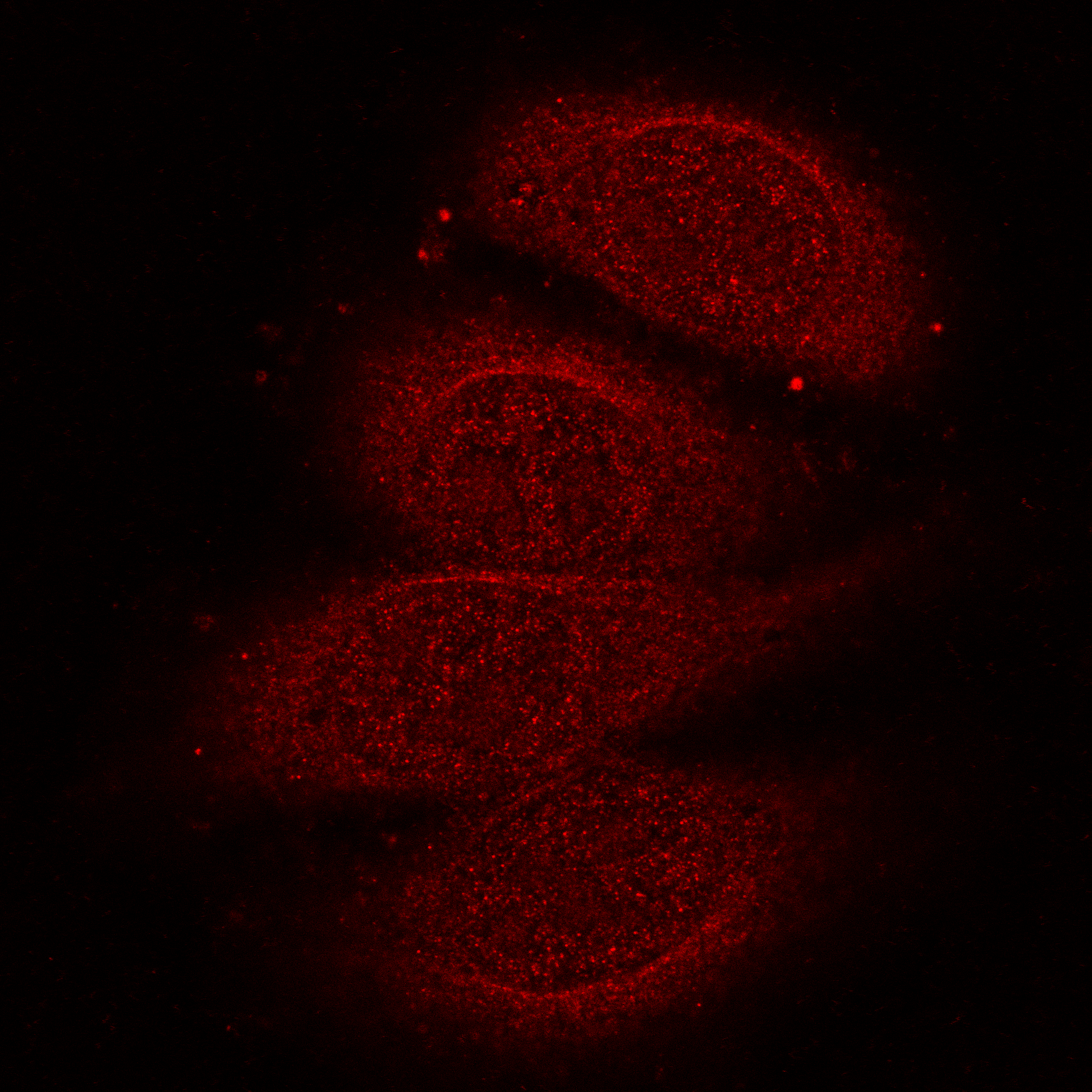

Supplement: Figure 5—source data 1. [file elife-87419-fig5-data1.zip › Figure 5-source data 1/5A/20210806-0907-1123-staining/20210907-15_Series001_ch02.tif]

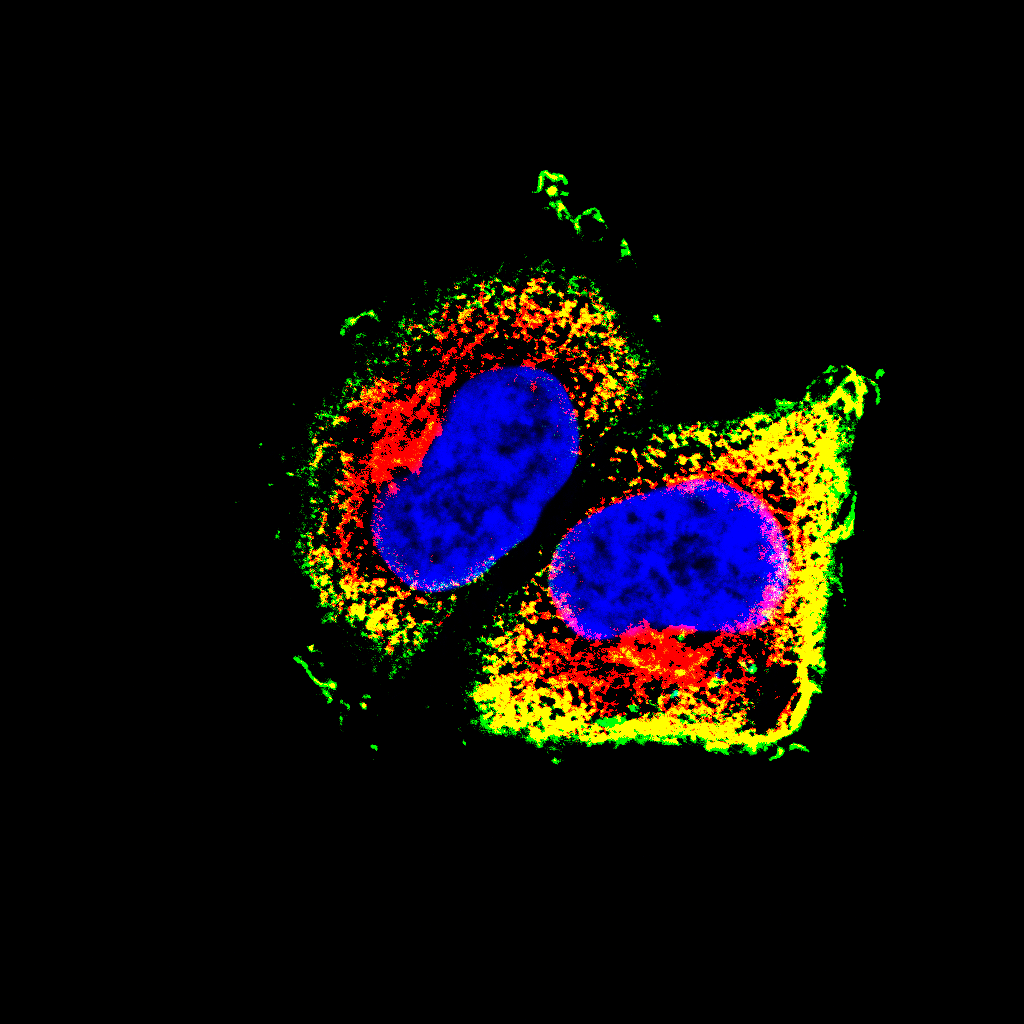

Supplement: Figure 5—source data 1. [file elife-87419-fig5-data1.zip › Figure 5-source data 1/5A/20210806-0907-1123-staining/20211123_Series021.tif]

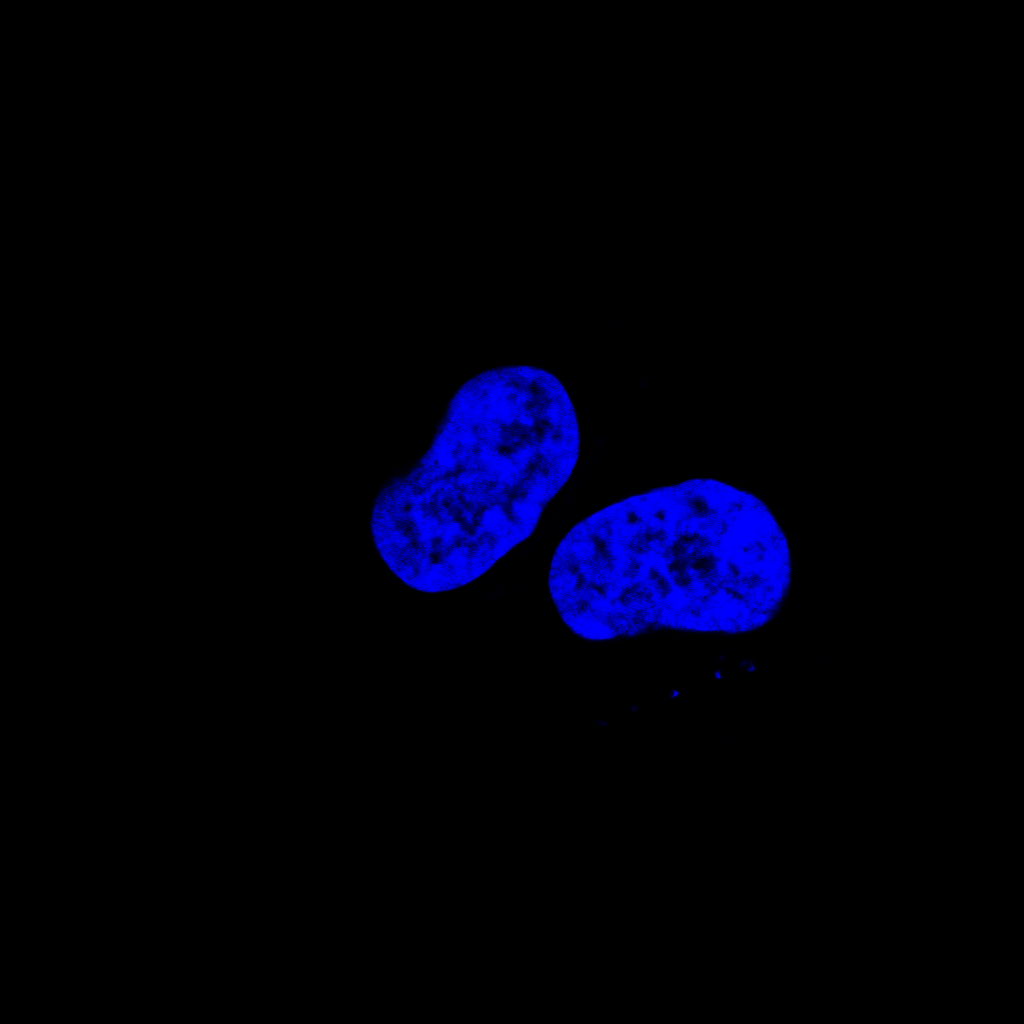

Supplement: Figure 5—source data 1. [file elife-87419-fig5-data1.zip › Figure 5-source data 1/5A/20210806-0907-1123-staining/20211123_Series021_ch00.tif]

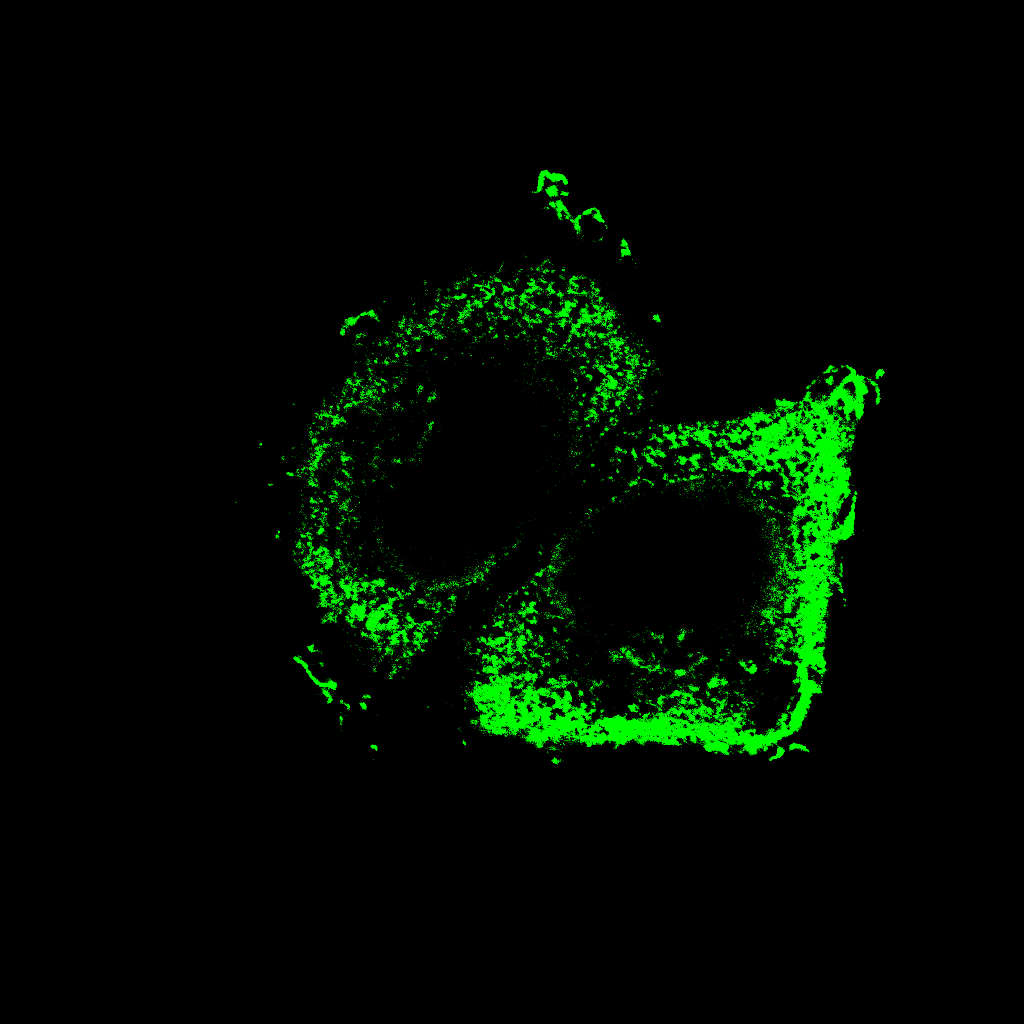

Supplement: Figure 5—source data 1. [file elife-87419-fig5-data1.zip › Figure 5-source data 1/5A/20210806-0907-1123-staining/20211123_Series021_ch01.tif]

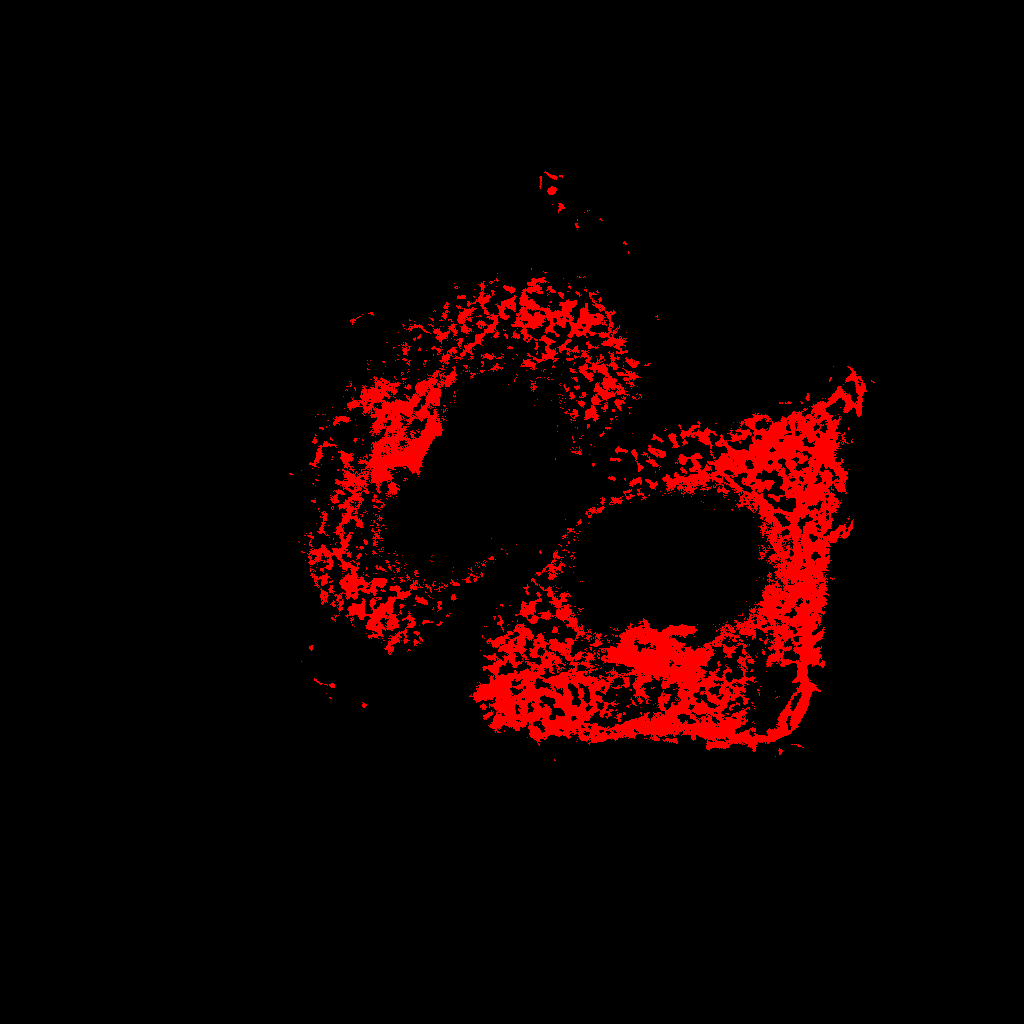

Supplement: Figure 5—source data 1. [file elife-87419-fig5-data1.zip › Figure 5-source data 1/5A/20210806-0907-1123-staining/20211123_Series021_ch02.tif]

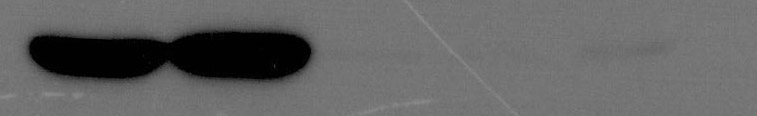

Supplement: Figure 5—source data 1. [file elife-87419-fig5-data1.zip › Figure 5-source data 1/5B/20210919-WB/0919-GAPDH.jpg]

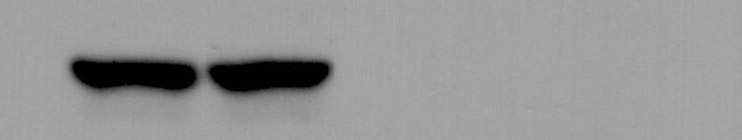

Supplement: Figure 5—source data 1. [file elife-87419-fig5-data1.zip › Figure 5-source data 1/5B/20210919-WB/12-1.jpg]

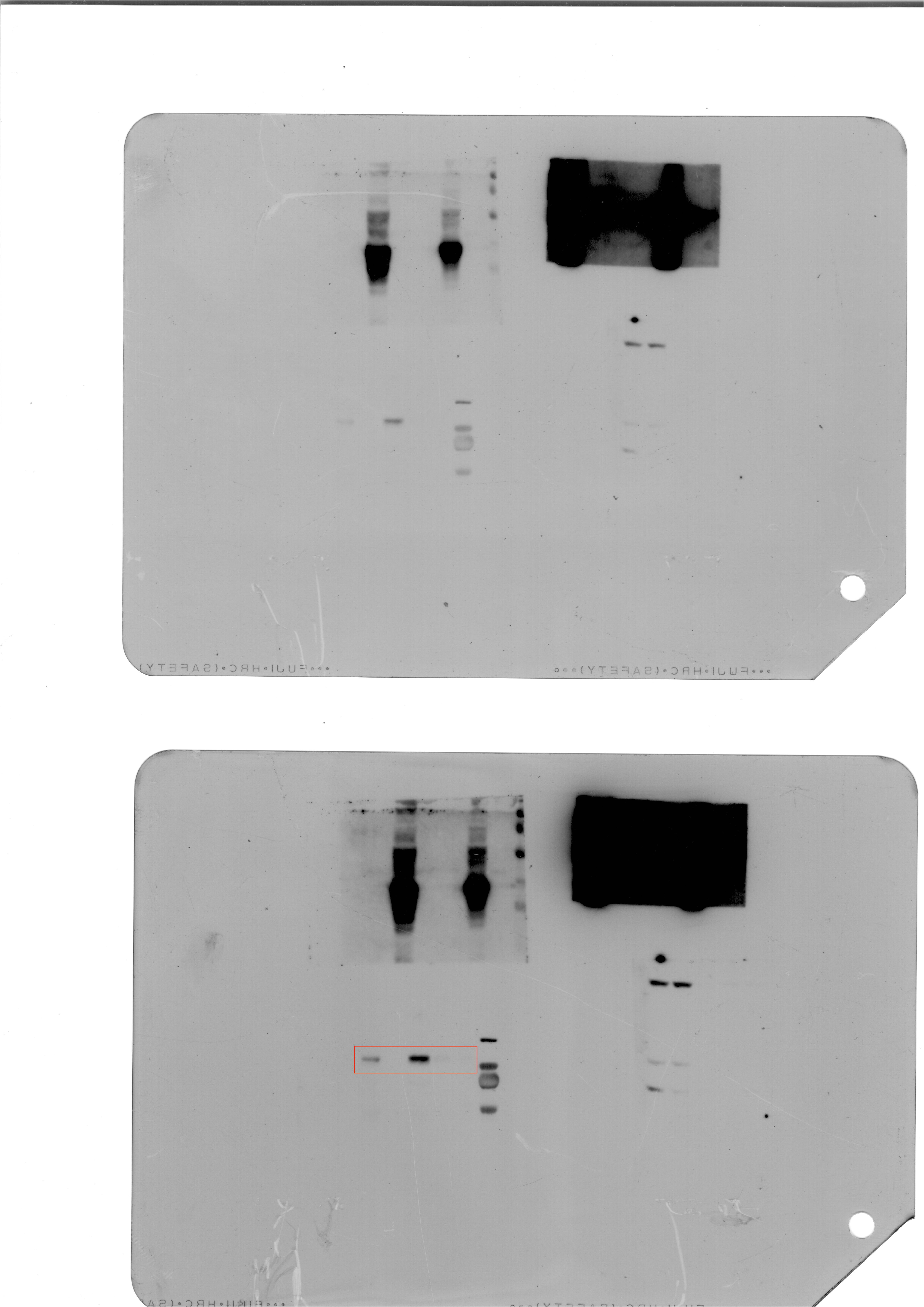

Supplement: Figure 5—source data 1. [file elife-87419-fig5-data1.zip › Figure 5-source data 1/5B/20210919-WB/2.jpg]

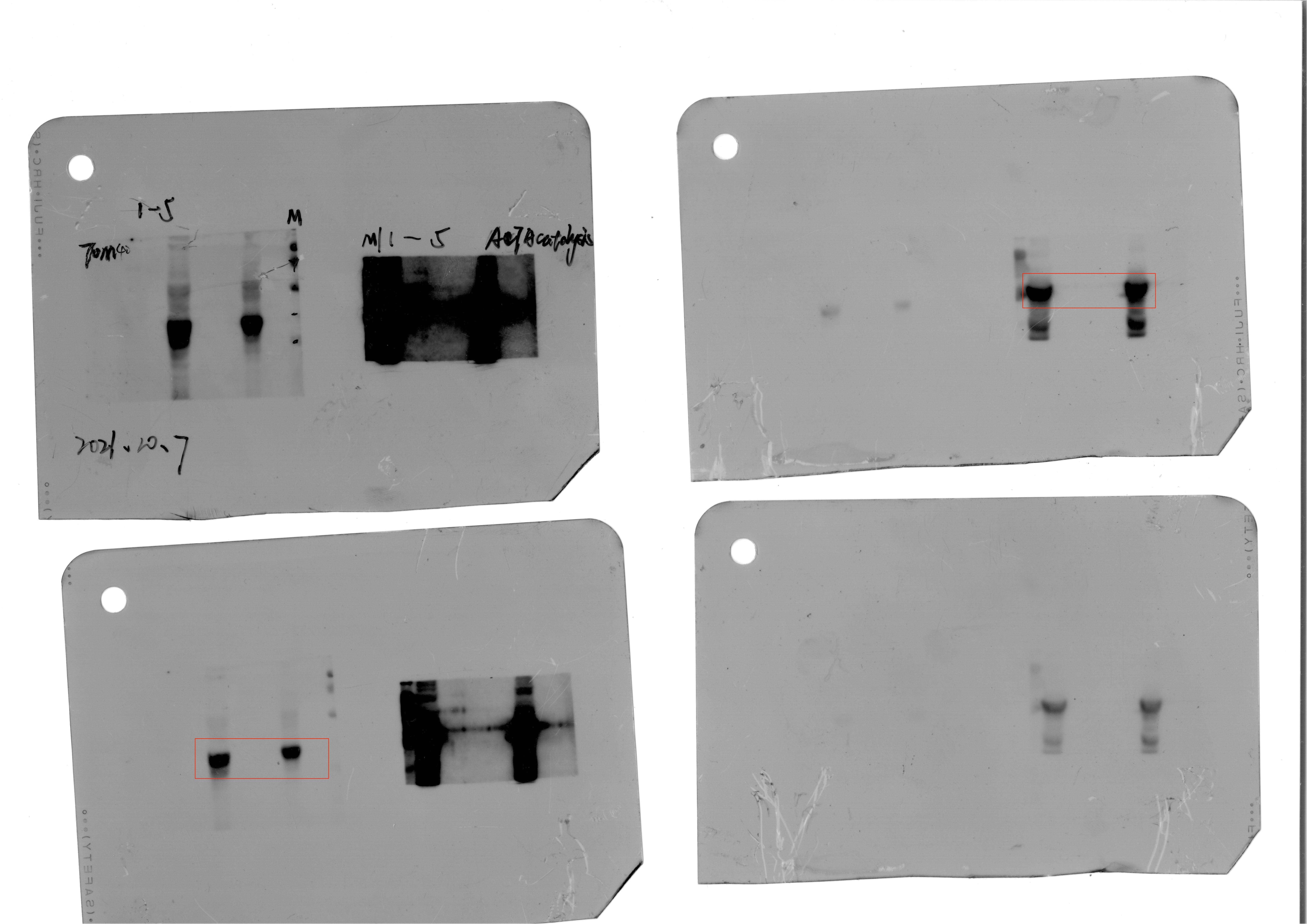

Supplement: Figure 5—source data 1. [file elife-87419-fig5-data1.zip › Figure 5-source data 1/5B/20210919-WB/3.jpg]

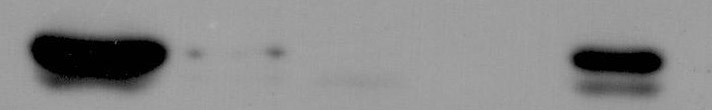

Supplement: Figure 5—source data 1. [file elife-87419-fig5-data1.zip › Figure 5-source data 1/5B/20210919-WB/8-1.jpg]

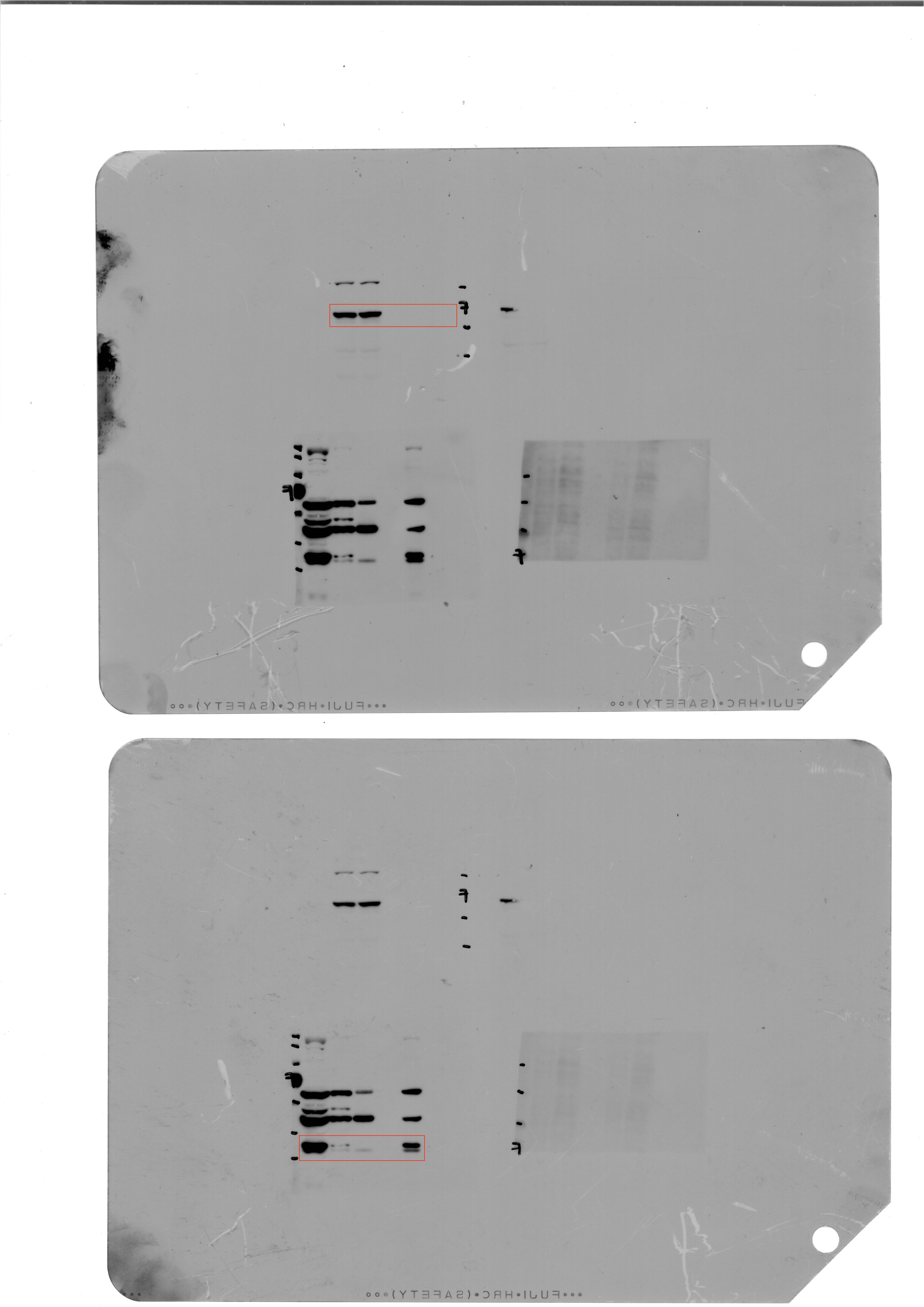

Supplement: Figure 5—source data 1. [file elife-87419-fig5-data1.zip › Figure 5-source data 1/5B/20210919-WB/ACOT8&12-1.jpg]

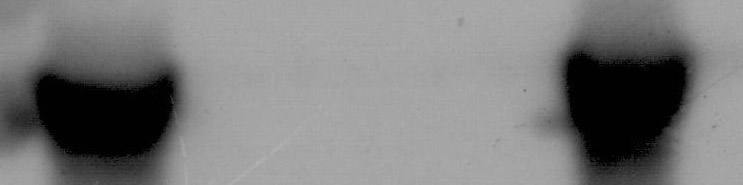

Supplement: Figure 5—source data 1. [file elife-87419-fig5-data1.zip › Figure 5-source data 1/5B/20210919-WB/cata-n.jpg]

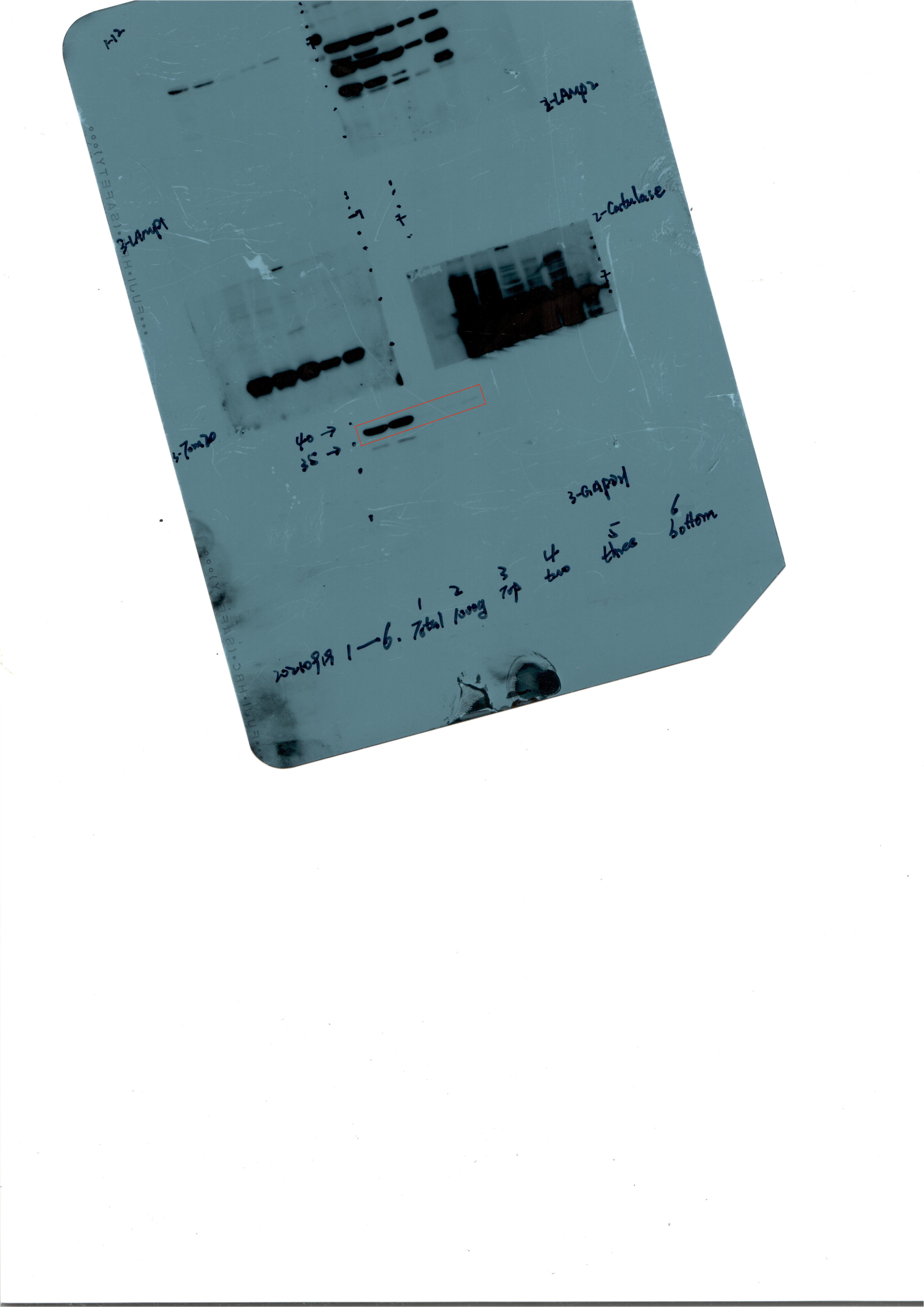

Supplement: Figure 5—source data 1. [file elife-87419-fig5-data1.zip › Figure 5-source data 1/5B/20210919-WB/GAPDH.jpg]

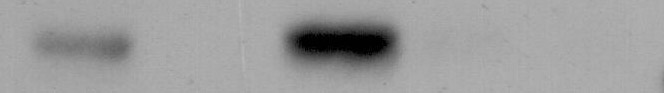

Supplement: Figure 5—source data 1. [file elife-87419-fig5-data1.zip › Figure 5-source data 1/5B/20210919-WB/LAMP2-n.jpg]

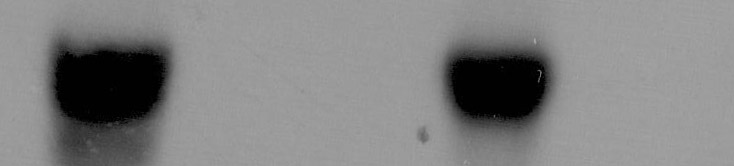

Supplement: Figure 5—source data 1. [file elife-87419-fig5-data1.zip › Figure 5-source data 1/5B/20210919-WB/tom40-n.jpg]

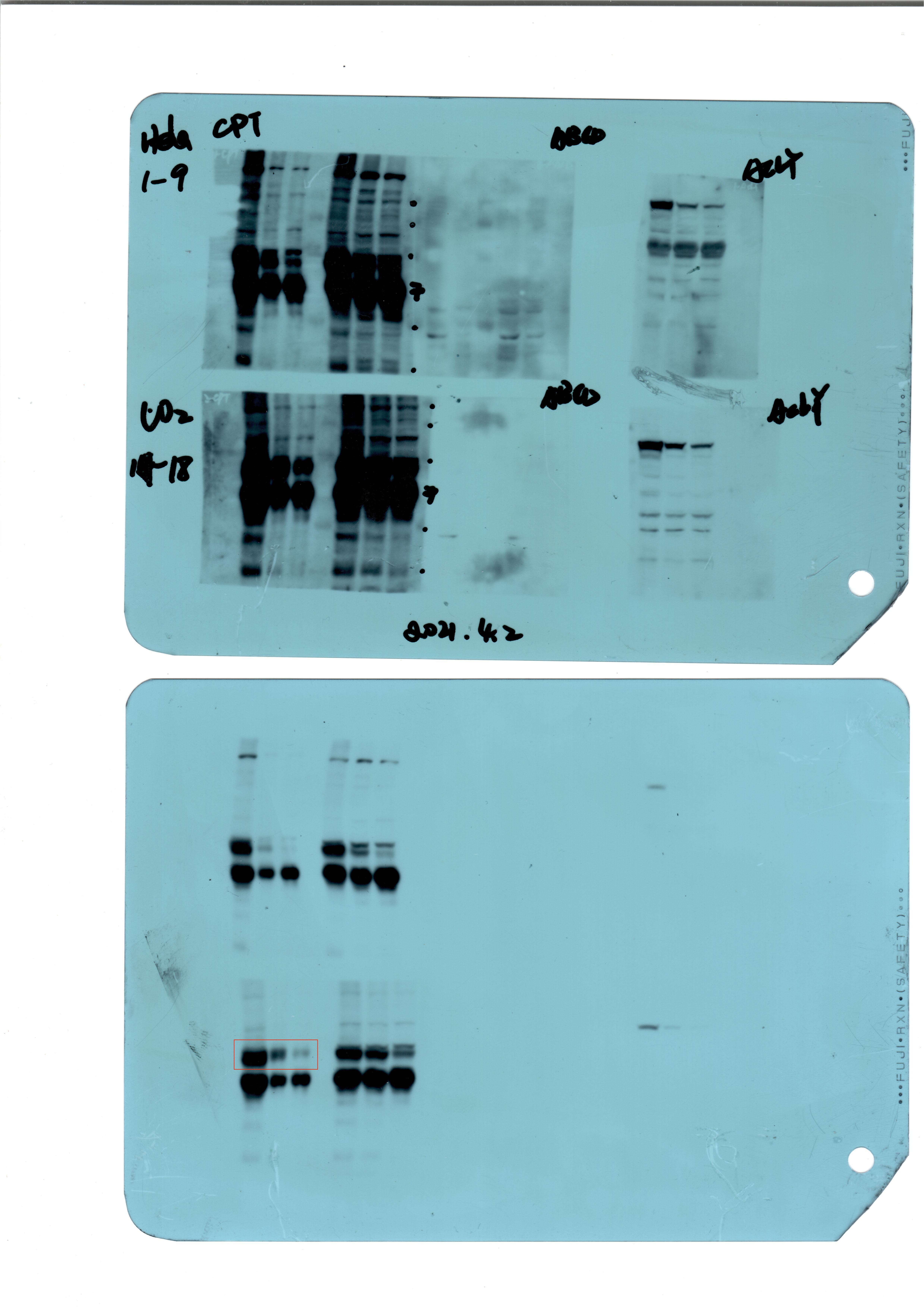

Supplement: Figure 5—source data 1. [file elife-87419-fig5-data1.zip › Figure 5-source data 1/5C-5G/20210402-WB/1.jpg]

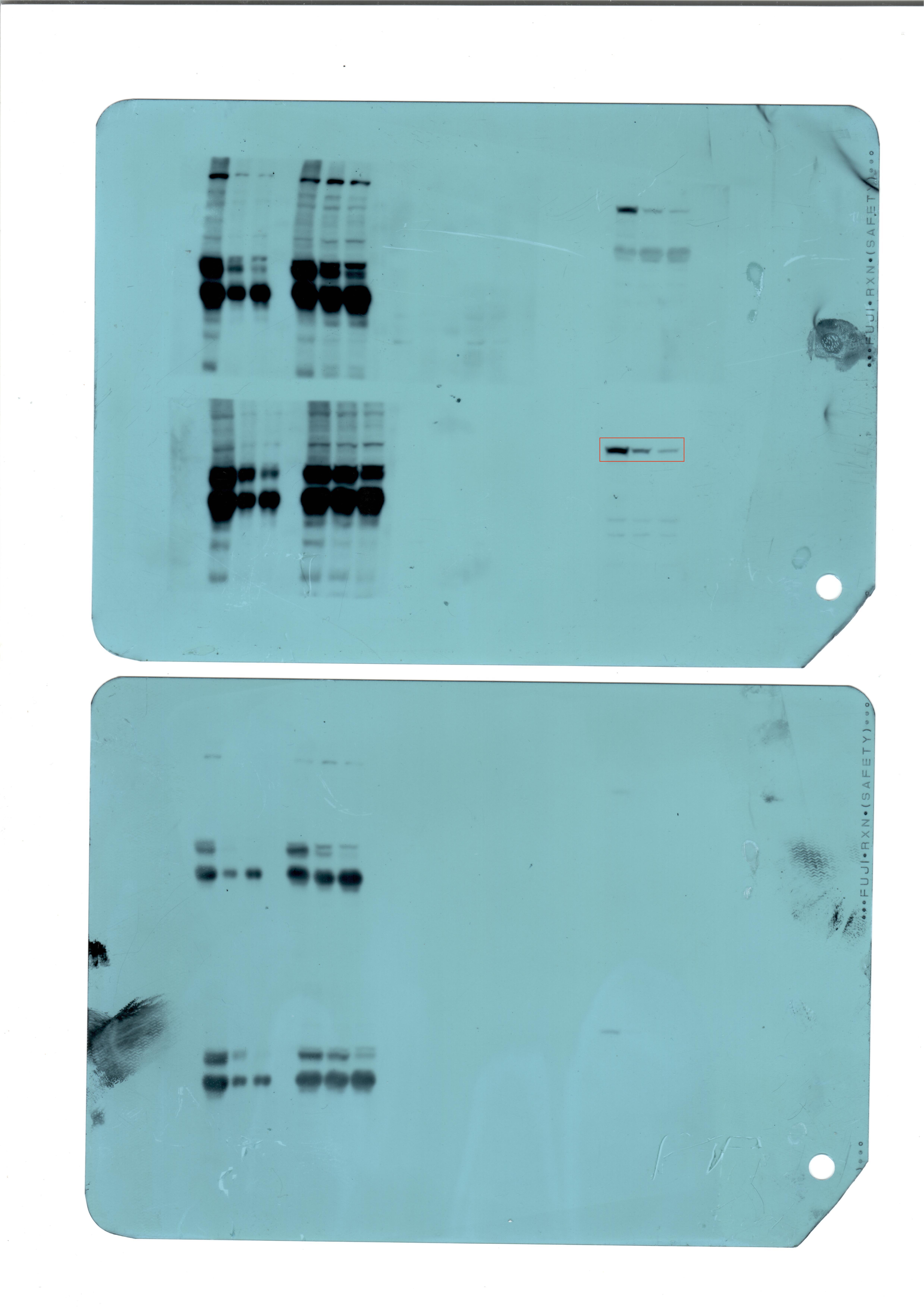

Supplement: Figure 5—source data 1. [file elife-87419-fig5-data1.zip › Figure 5-source data 1/5C-5G/20210402-WB/2.jpg]

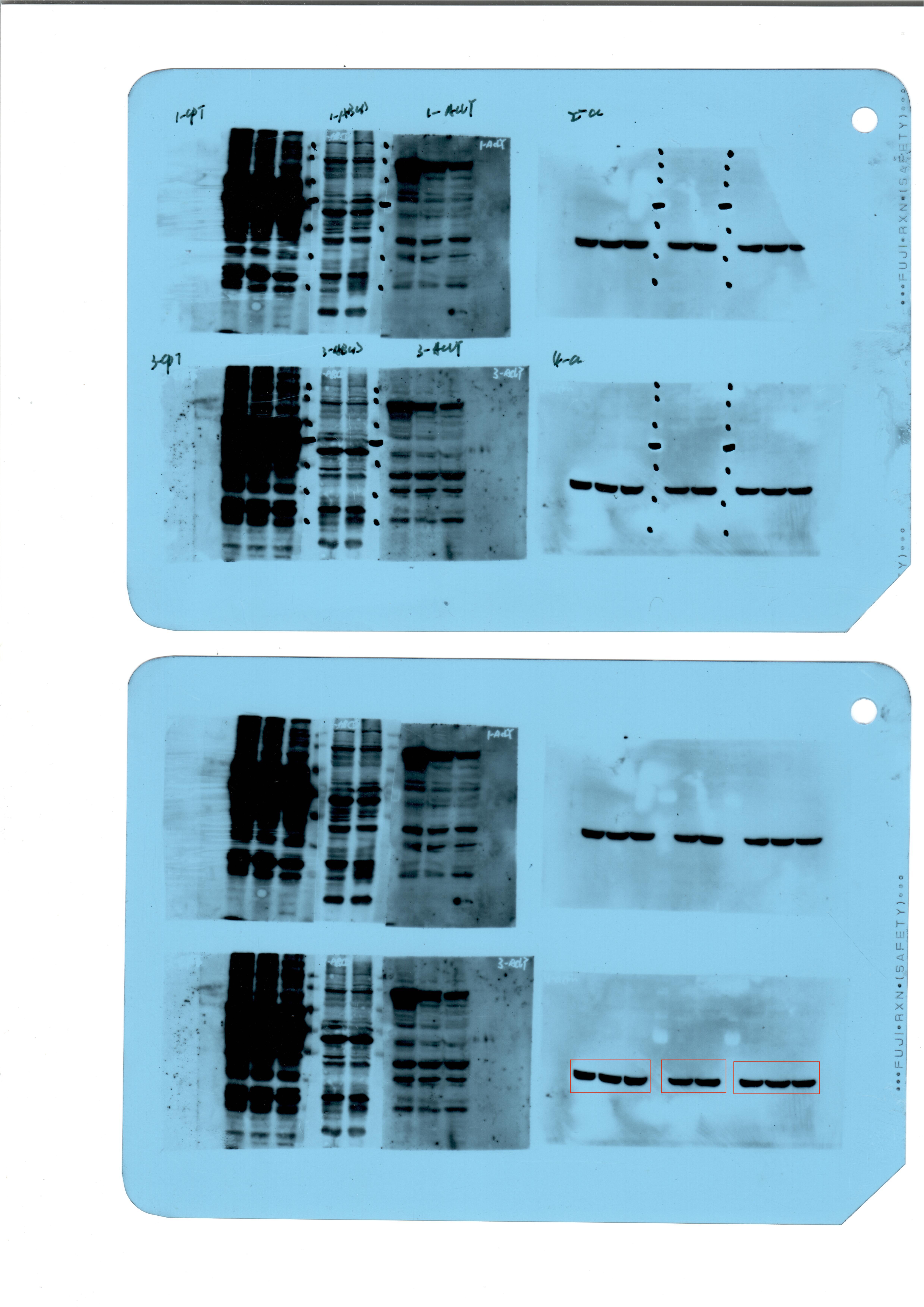

Supplement: Figure 5—source data 1. [file elife-87419-fig5-data1.zip › Figure 5-source data 1/5C-5G/20210402-WB/4.jpg]

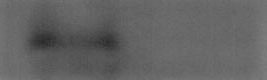

Supplement: Figure 5—source data 1. [file elife-87419-fig5-data1.zip › Figure 5-source data 1/5C-5G/20210402-WB/abcd1-LO2 .jpg]

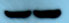

Supplement: Figure 5—source data 1. [file elife-87419-fig5-data1.zip › Figure 5-source data 1/5C-5G/20210402-WB/abcd1-LO2-actin.jpg]

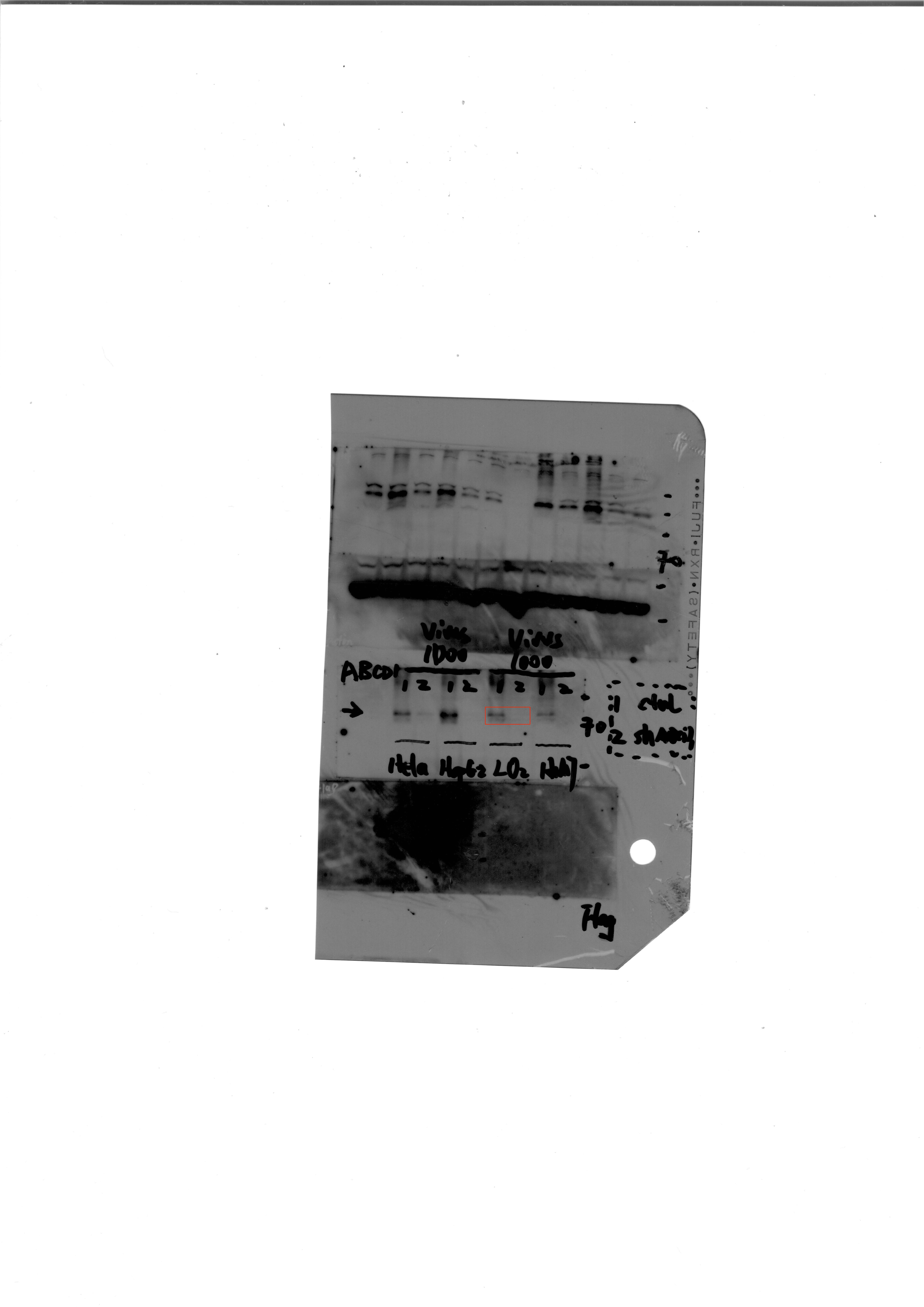

Supplement: Figure 5—source data 1. [file elife-87419-fig5-data1.zip › Figure 5-source data 1/5C-5G/20210402-WB/abcd1.jpg]

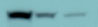

Supplement: Figure 5—source data 1. [file elife-87419-fig5-data1.zip › Figure 5-source data 1/5C-5G/20210402-WB/acly-lo2.jpg]

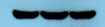

Supplement: Figure 5—source data 1. [file elife-87419-fig5-data1.zip › Figure 5-source data 1/5C-5G/20210402-WB/actin-lo2.jpg]

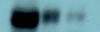

Supplement: Figure 5—source data 1. [file elife-87419-fig5-data1.zip › Figure 5-source data 1/5C-5G/20210402-WB/cpt-lo2.jpg]

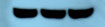

Supplement: Figure 5—source data 1. [file elife-87419-fig5-data1.zip › Figure 5-source data 1/5C-5G/20210402-WB/cpt1-actin-lo2.jpg]

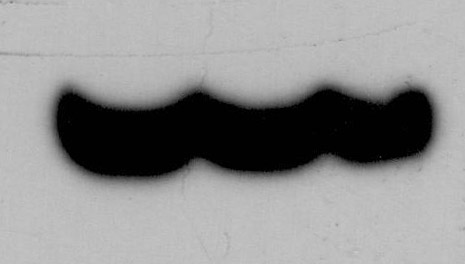

Supplement: Figure 7—source data 1. [file elife-87419-fig7-data1.zip › Figure 7-source data 1/7DE/20210921-WB/0921-12-actin.jpg]

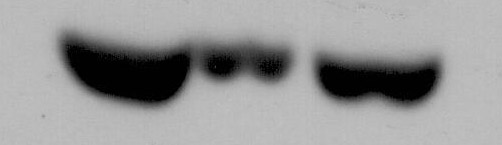

Supplement: Figure 7—source data 1. [file elife-87419-fig7-data1.zip › Figure 7-source data 1/7DE/20210921-WB/0921-12-HMGCS2.jpg]

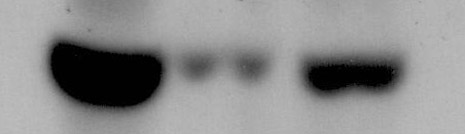

Supplement: Figure 7—source data 1. [file elife-87419-fig7-data1.zip › Figure 7-source data 1/7DE/20210921-WB/0921-12.jpg]

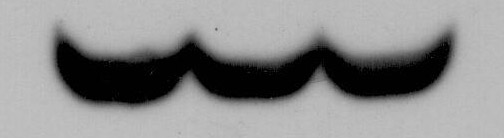

Supplement: Figure 7—source data 1. [file elife-87419-fig7-data1.zip › Figure 7-source data 1/7DE/20210921-WB/0921-8-actin.jpg]

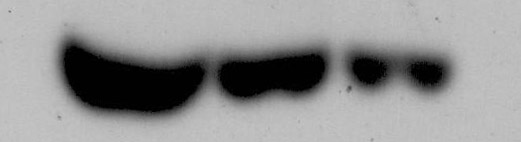

Supplement: Figure 7—source data 1. [file elife-87419-fig7-data1.zip › Figure 7-source data 1/7DE/20210921-WB/0921-8-HMGCS2.jpg]

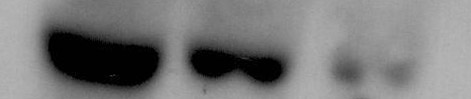

Supplement: Figure 7—source data 1. [file elife-87419-fig7-data1.zip › Figure 7-source data 1/7DE/20210921-WB/0921-8.jpg]

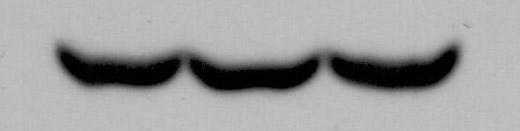

Supplement: Figure 7—source data 1. [file elife-87419-fig7-data1.zip › Figure 7-source data 1/7DE/20210921-WB/0921-actin2.jpg]

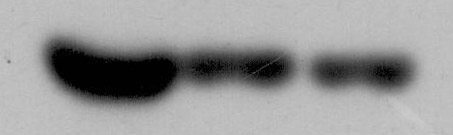

Supplement: Figure 7—source data 1. [file elife-87419-fig7-data1.zip › Figure 7-source data 1/7DE/20210921-WB/0922-HMGCS2.jpg]

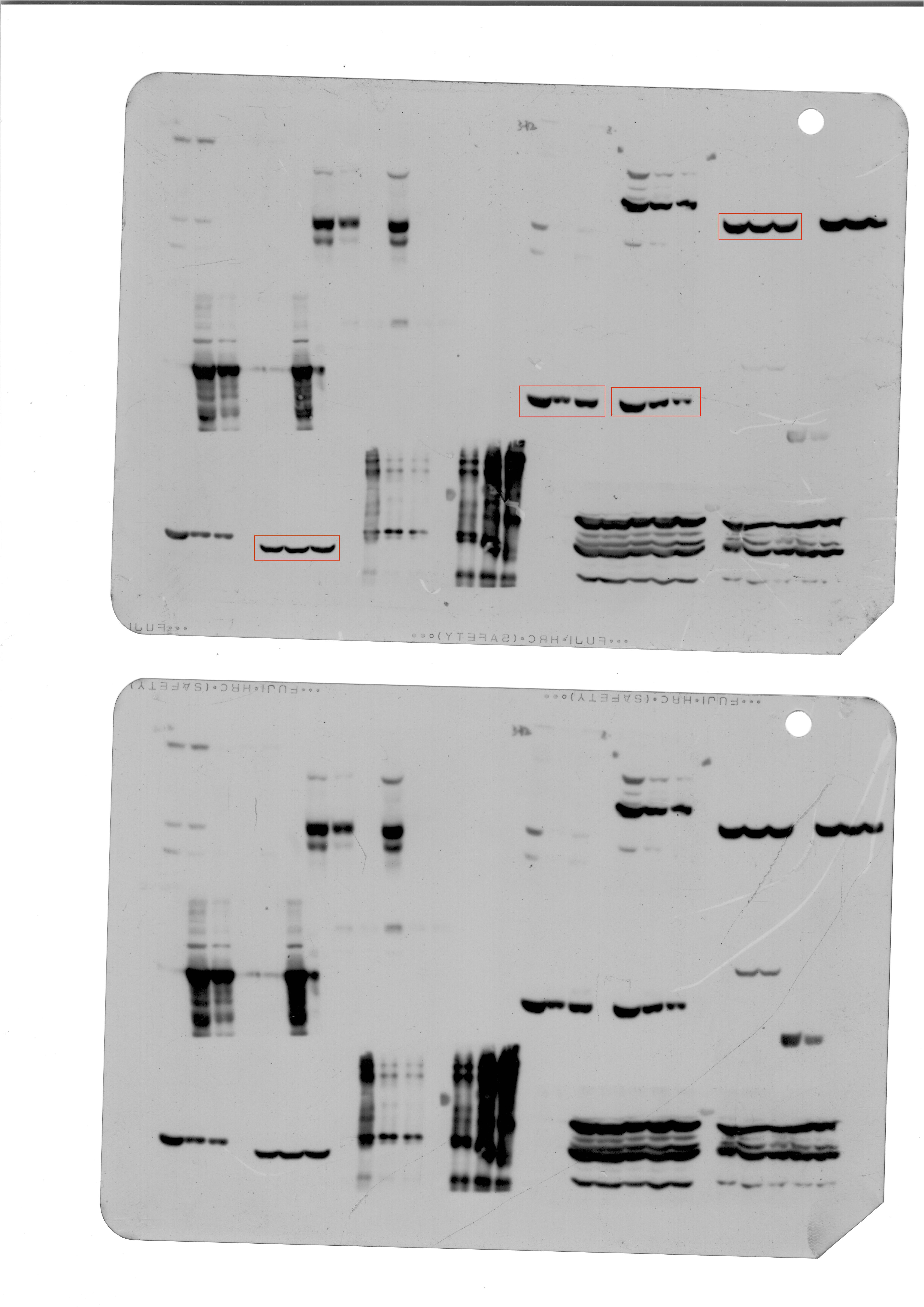

Supplement: Figure 7—source data 1. [file elife-87419-fig7-data1.zip › Figure 7-source data 1/7DE/20210921-WB/2.jpg]

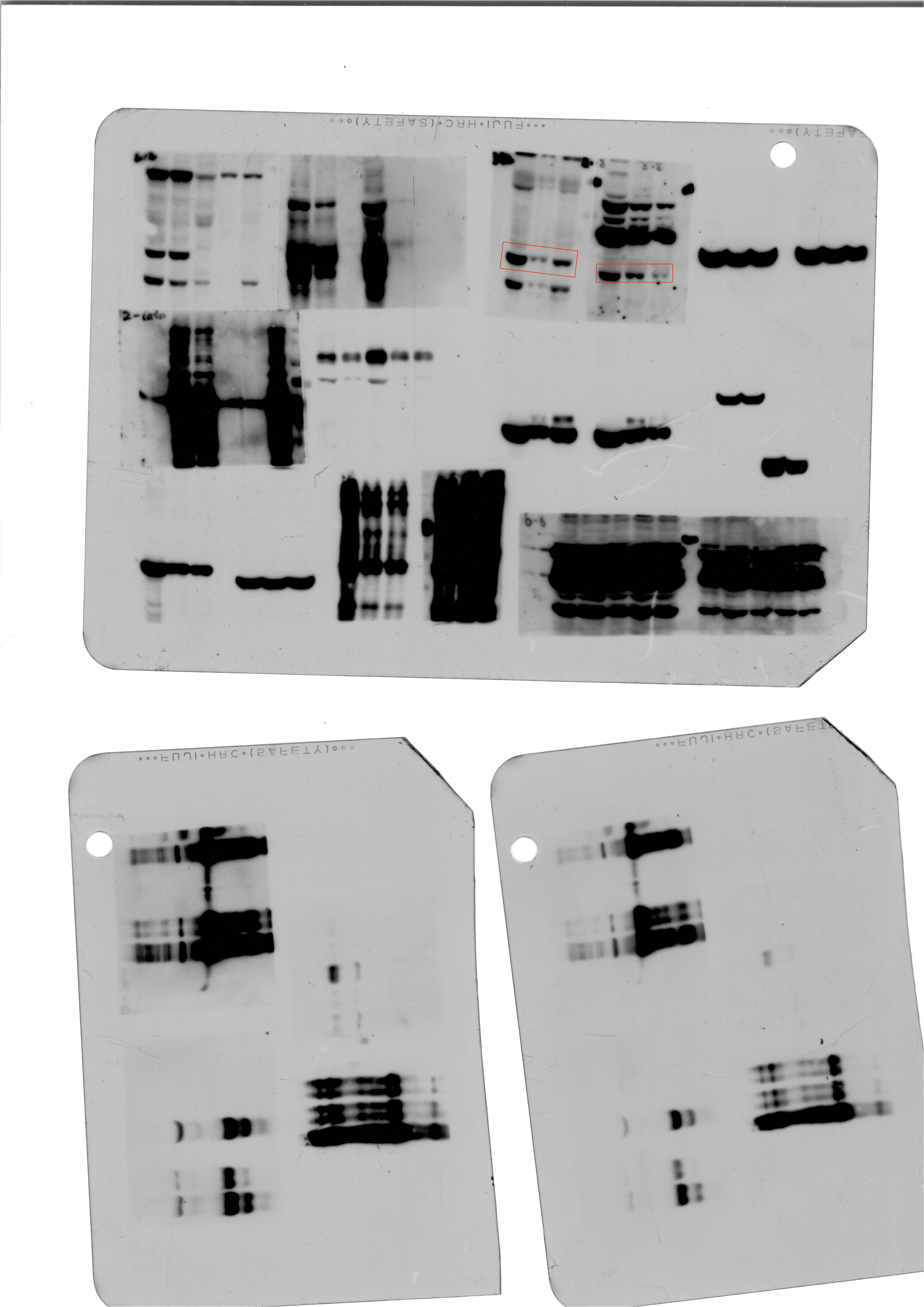

Supplement: Figure 7—source data 1. [file elife-87419-fig7-data1.zip › Figure 7-source data 1/7DE/20210921-WB/3.jpg]

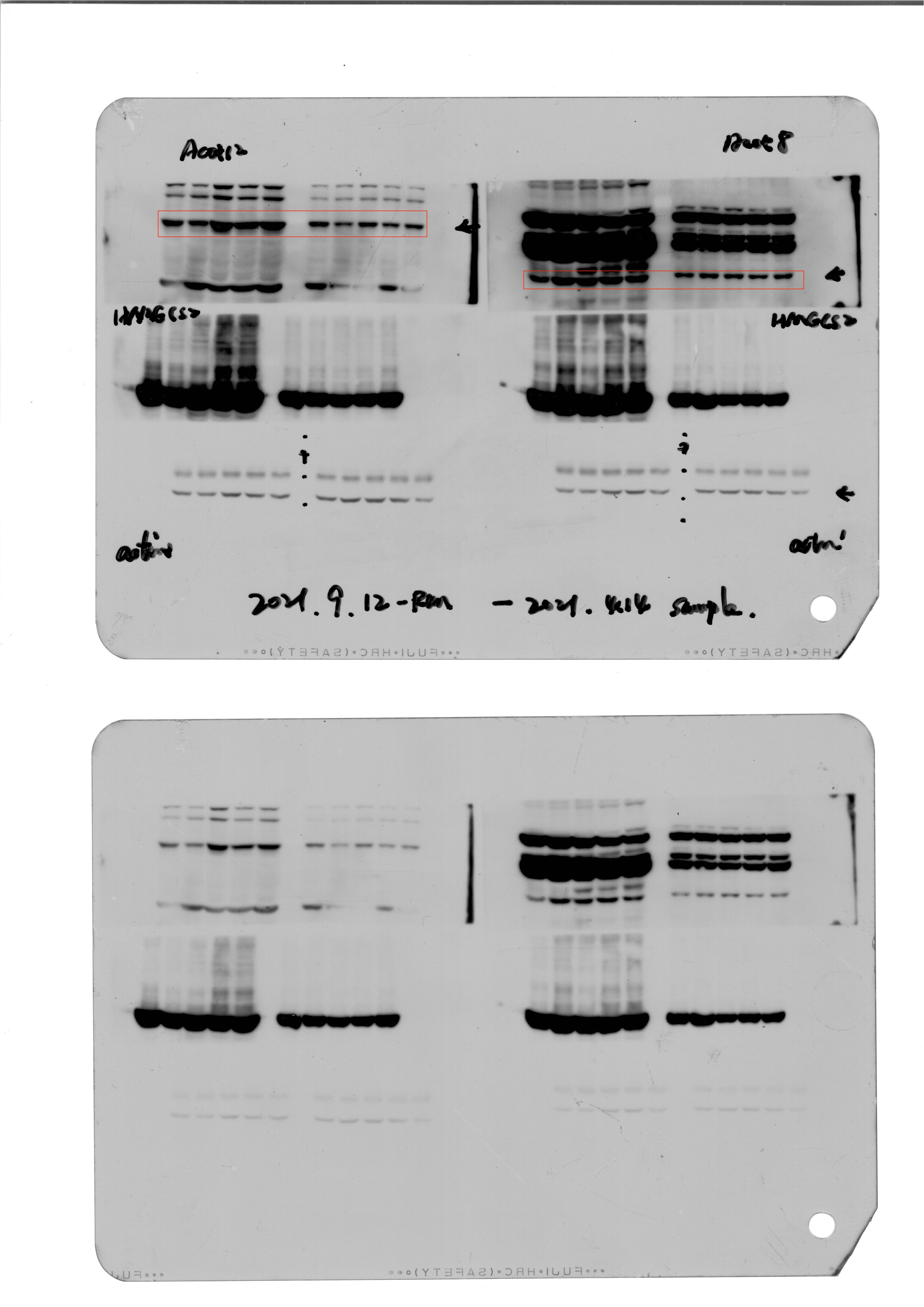

Supplement: Figure 7—source data 1. [file elife-87419-fig7-data1.zip › Figure 7-source data 1/7FG/20210912-0414-WB/1.jpg]

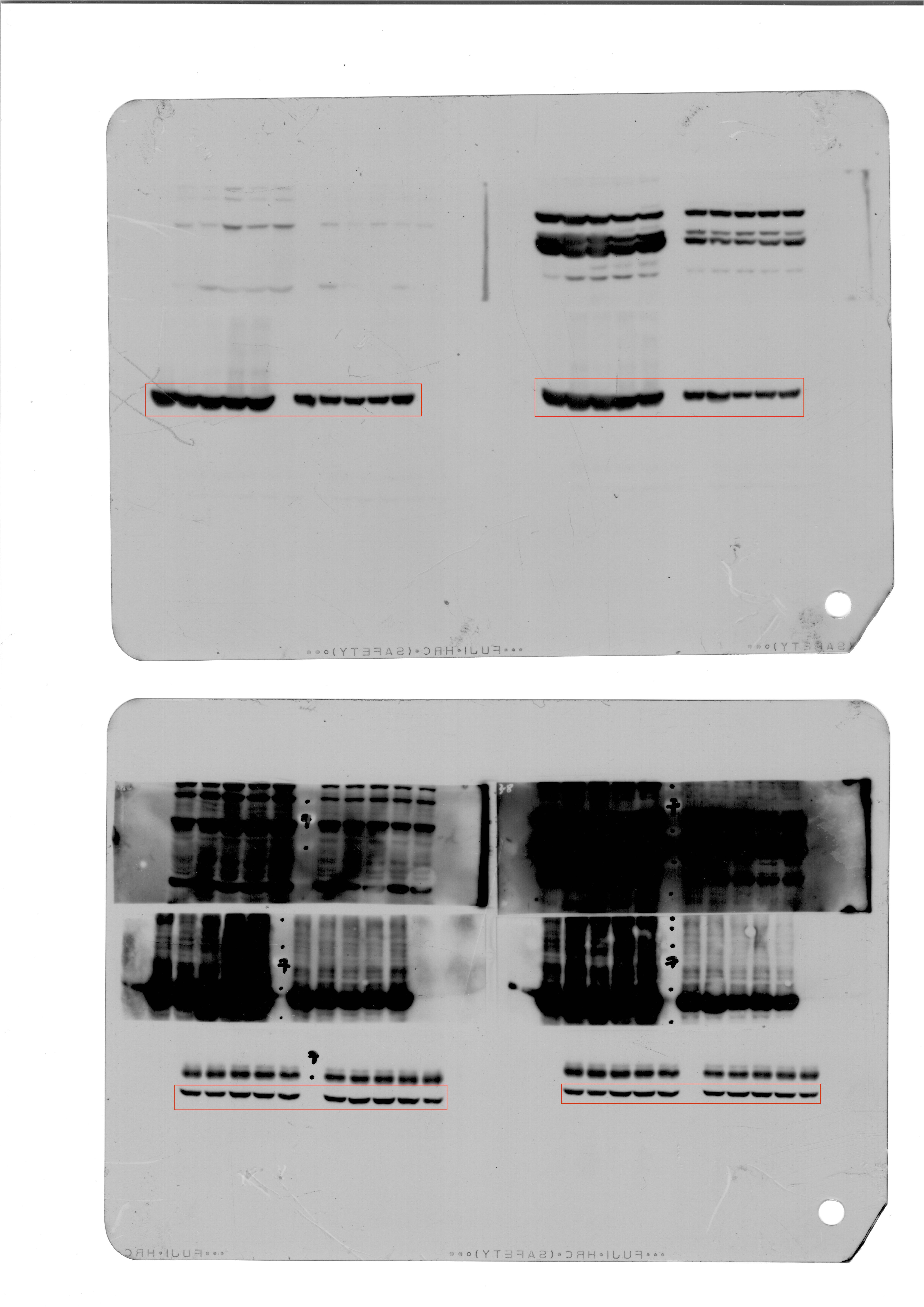

Supplement: Figure 7—source data 1. [file elife-87419-fig7-data1.zip › Figure 7-source data 1/7FG/20210912-0414-WB/2.jpg]

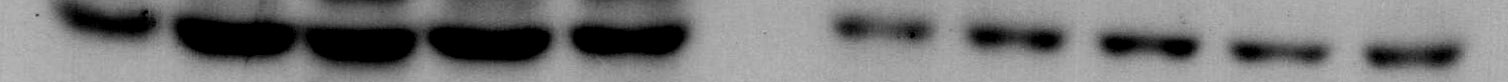

Supplement: Figure 7—source data 1. [file elife-87419-fig7-data1.zip › Figure 7-source data 1/7FG/20210912-0414-WB/8-n.jpg]

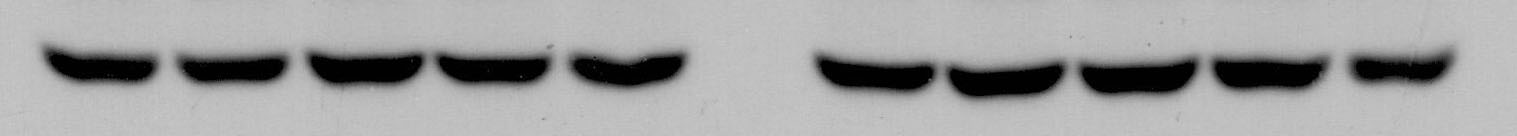

Supplement: Figure 7—source data 1. [file elife-87419-fig7-data1.zip › Figure 7-source data 1/7FG/20210912-0414-WB/sh12-actin.jpg]

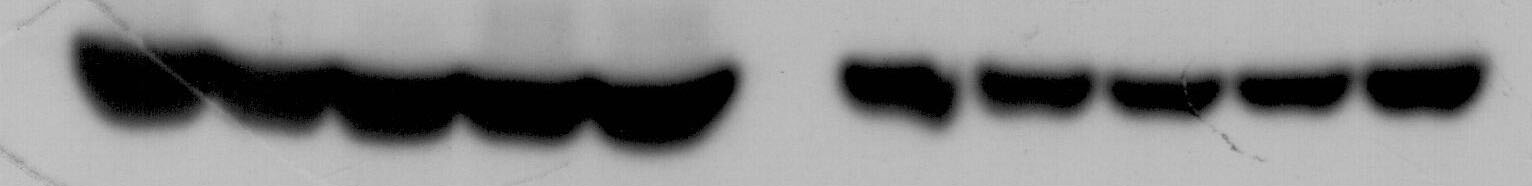

Supplement: Figure 7—source data 1. [file elife-87419-fig7-data1.zip › Figure 7-source data 1/7FG/20210912-0414-WB/sh12-hmgcs2-2.jpg]

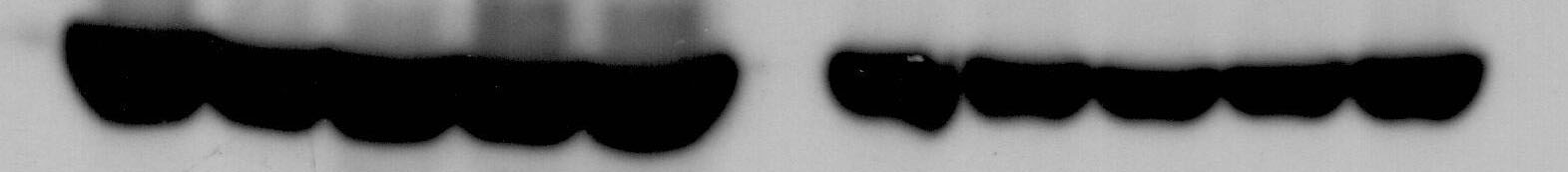

Supplement: Figure 7—source data 1. [file elife-87419-fig7-data1.zip › Figure 7-source data 1/7FG/20210912-0414-WB/sh12-hmgcs2.jpg]

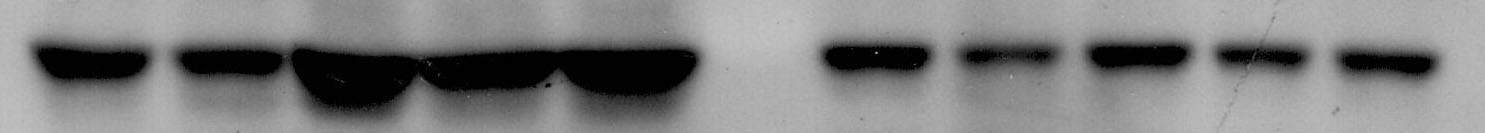

Supplement: Figure 7—source data 1. [file elife-87419-fig7-data1.zip › Figure 7-source data 1/7FG/20210912-0414-WB/sh12.jpg]

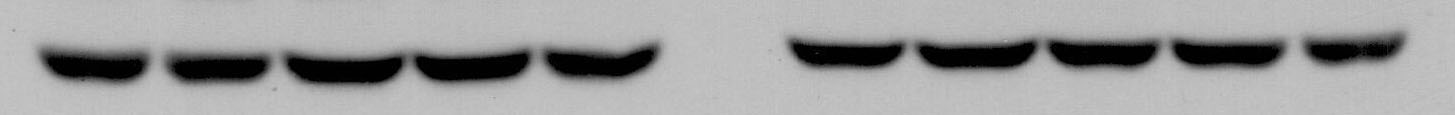

Supplement: Figure 7—source data 1. [file elife-87419-fig7-data1.zip › Figure 7-source data 1/7FG/20210912-0414-WB/sh8-actin.jpg]

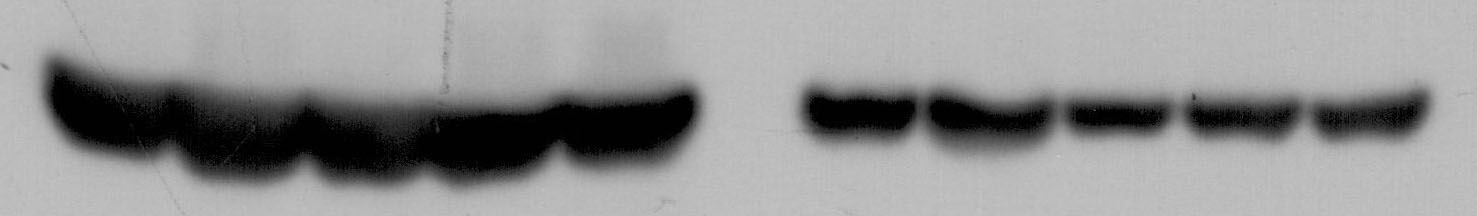

Supplement: Figure 7—source data 1. [file elife-87419-fig7-data1.zip › Figure 7-source data 1/7FG/20210912-0414-WB/sh8-hmgcs2-2.jpg]
