# Supplementary material for: Hepatic conversion of acetyl-CoA to acetate plays crucial roles in energy stress
Source: eLife. 2023 Oct 30;12:RP87419. doi: 10.7554/eLife.87419 (PMC10615369; doi:10.7554/eLife.87419)
Supplement: Figure 7—source data 1. [file elife-87419-fig7-data1.zip › Figure 7-source data 1.docx]

**Figure 7—source data 1**

Complete, unedited immunoblots, as well as immunoblots including sample and band identification, are provided for the immunoblots presented in Figure 7.
