# Supplementary figures and images for: Hepatic conversion of acetyl-CoA to acetate plays crucial roles in energy stress (part 2 of 2)
Source: eLife. 2023 Oct 30;12:RP87419. doi: 10.7554/eLife.87419 (PMC10615369; doi:10.7554/eLife.87419)

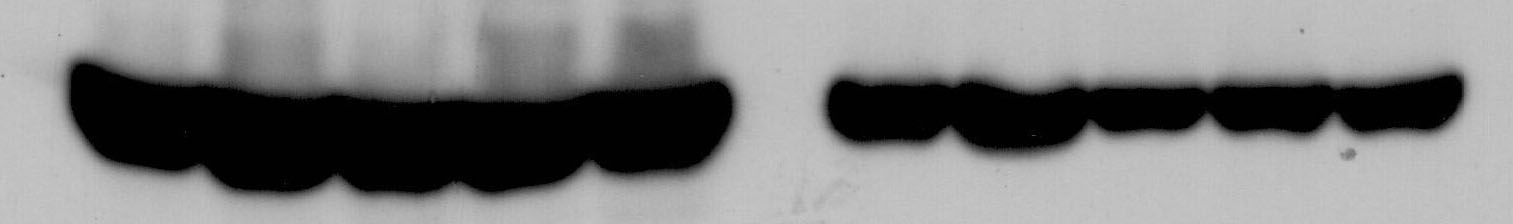

Supplement: Figure 7—source data 1. [file elife-87419-fig7-data1.zip › Figure 7-source data 1/7FG/20210912-0414-WB/sh8-hmgcs2-3.jpg]

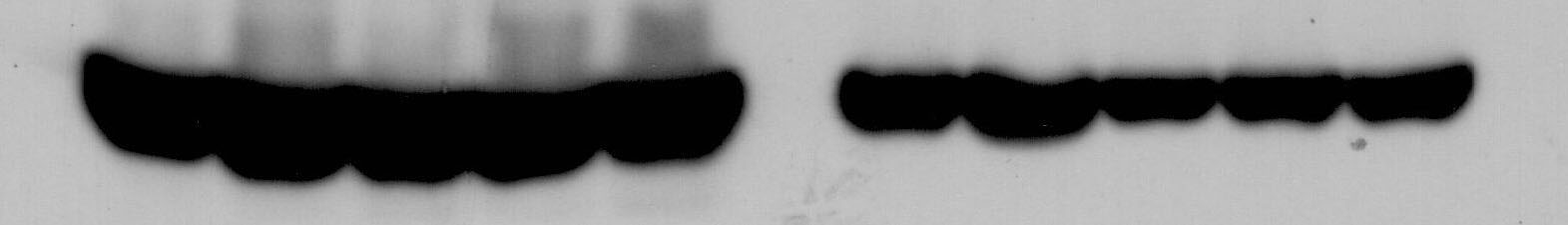

Supplement: Figure 7—source data 1. [file elife-87419-fig7-data1.zip › Figure 7-source data 1/7FG/20210912-0414-WB/sh8-hmgcs2.jpg]

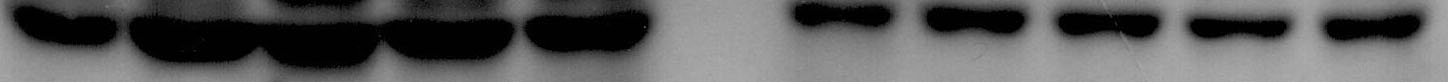

Supplement: Figure 7—source data 1. [file elife-87419-fig7-data1.zip › Figure 7-source data 1/7FG/20210912-0414-WB/sh8.jpg]

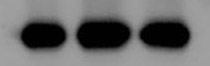

Supplement: Figure 7—source data 1. [file elife-87419-fig7-data1.zip › Figure 7-source data 1/7H/20211108-WB/Acetyl-1.jpg]

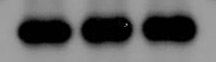

Supplement: Figure 7—source data 1. [file elife-87419-fig7-data1.zip › Figure 7-source data 1/7H/20211108-WB/Acetyl-2.jpg]

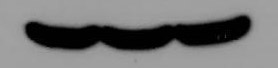

Supplement: Figure 7—source data 1. [file elife-87419-fig7-data1.zip › Figure 7-source data 1/7H/20211108-WB/actin.jpg]

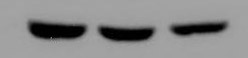

Supplement: Figure 7—source data 1. [file elife-87419-fig7-data1.zip › Figure 7-source data 1/7H/20211108-WB/HMGCS.jpg]

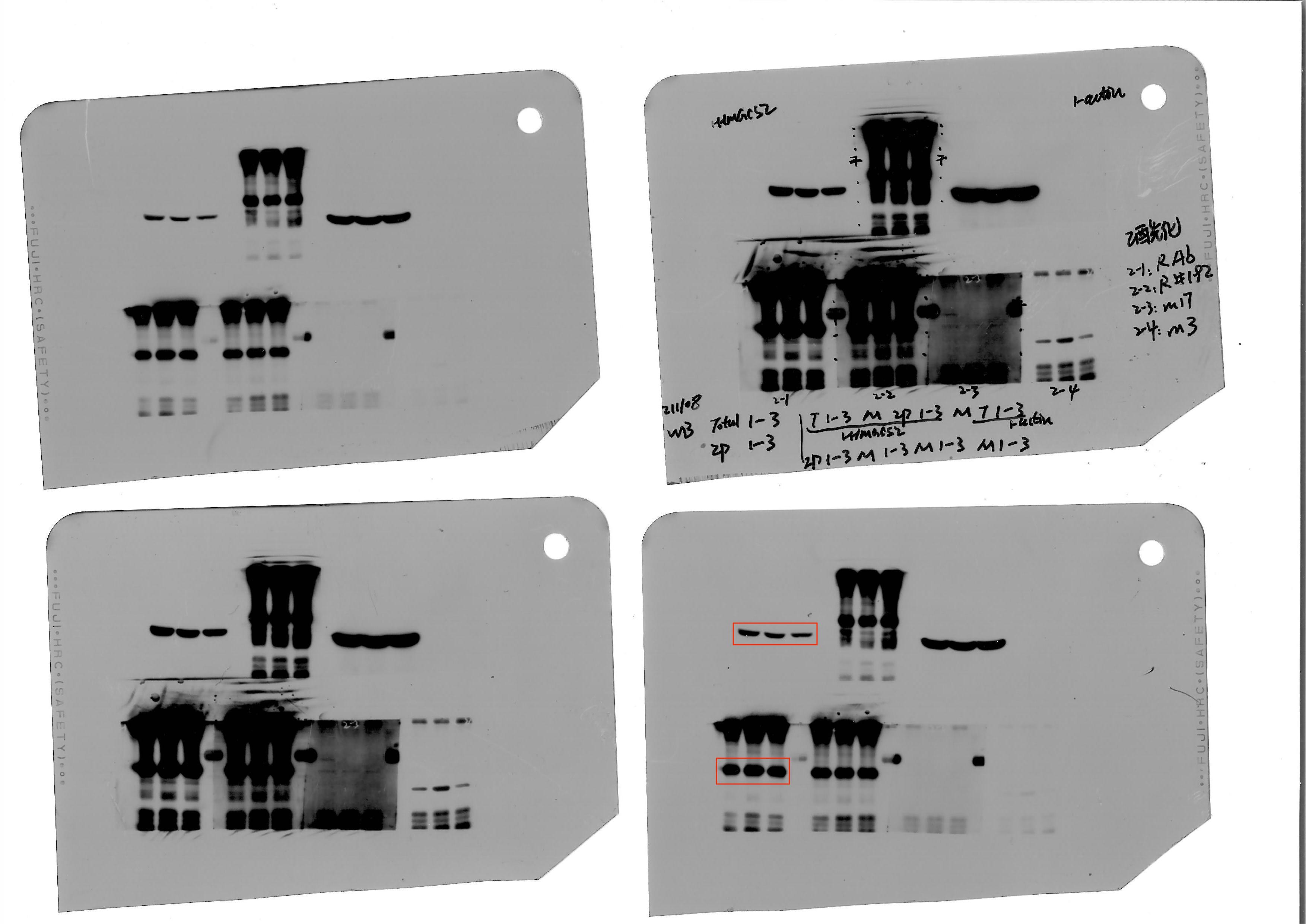

Supplement: Figure 7—source data 1. [file elife-87419-fig7-data1.zip › Figure 7-source data 1/7H/20211108-WB/Scan.jpg]
